# Light-directed trapping of metastable intermediates in a self-assembly process

**DOI:** 10.1038/s41467-020-20172-6

**Published:** 2020-12-07

**Authors:** Joonsik Seo, Joonyoung F. Joung, Sungnam Park, Young Ji Son, Jaegeun Noh, Jong-Man Kim

**Affiliations:** 1 Department of Chemical Engineering, Hanyang University, Seoul, 04763 Korea; 2 Department of Chemistry and Research Institute for Natural Science, Korea University, Seoul, 02841 Korea; 3 Department of Chemistry, Hanyang University, Seoul, 04763 Korea; 4 Institute of Nano Science and Technology, Hanyang University, Seoul, 04763 Korea

**Keywords:** Self-assembly, Self-assembly, Structural properties

## Abstract

Self-assembly is a dynamic process that often takes place through a stepwise pathway involving formation of kinetically favored metastable intermediates prior to generation of a thermodynamically preferred supramolecular framework. Although trapping intermediates in these pathways can provide significant information about both their nature and the overall self-assembly process, it is a challenging venture without altering temperature, concentrations, chemical compositions and morphologies. Herein, we report a highly efficient and potentially general method for “trapping” metastable intermediates in self-assembly processes that is based on a photopolymerization strategy. By employing a chiral perylene-diimide possessing a diacetylene containing an alkyl chain, we demonstrated that the metastable intermediates, including nanoribbons, nanocoils and nanohelices, can be effectively trapped by using UV promoted polymerization before they form thermodynamic tubular structures. The strategy developed in this study should be applicable to naturally and synthetically abundant alkyl chain containing self-assembling systems.

## Abstract (web summary)

Knowledge about kinetically favored intermediate states in self-assembly processes can provide information about the self-assembly process but trapping these states without changing the reaction conditions is challenging. Here, the authors report a method for trapping metastable intermediates in self-assembly processes that is based on a photopolymerization strategy.

## Introduction

Self-assembly, a spontaneous process that leads to formation of a functional supramolecule from building blocks, is elegantly utilized in nature for construction of a variety of complex biological structures. Chemists and material scientists have extensively investigated nature’s self-assembly principles, to devise methods to fabricate diverse biomimetic nanomaterials^1–20^. Self-assembly is a dynamic process often occurring through pathways in which kinetically favored metastable intermediates are first generated and then dissociate and reorganize to form thermodynamically more stable molecular assemblies^21–29^. Although information about the metastable intermediates can provide an important understanding of the nature of the self-assembly pathway, “trapping” of these intermediates without altering temperature, concentration, solvent/chemical composition, and morphology is difficult.

Elegant methods that utilize a ring-opening metathesis polymerization (ROMP)^30^ and a Click chemistry^31^ to lock the structures of metastable intermediates in self-assembly pathways have been described recently. A Grubbs catalyst-promoted ROMP of norbornene-functionalized hexabenzocoronene derivatives was found to effectively trap nanocoil intermediates^30^. A Click reaction between terminal alkyne-containing building blocks and azide cross-linkers resulted in locking of a perylene diimide (PDI) supramolecular polymer^31^. However, these methods lack generality, because they require the use of catalysts that may interfere with the self-assembly process. In addition, a substantial amount of time (greater than several hours) is required for the catalyst-promoted ROMP or Click reaction to take place in solution. Thus, fast trapping of the metastable intermediates is difficult to achieve using these methods.

It is well-known that ultraviolet (UV) irradiation of self-assembled diacetylene (DA) supramolecules leads to polymerization through crosslinking of the DA units via formation of conjugated ene-yne bonds to generate structurally stable polydiacetylenes (PDAs)^32–37^. In addition, these photopolymerization reactions occur with minimum rearrangement of the DA building blocks and thus the nanoscale morphologies of the PDA supramolecules are almost the same as those of the corresponding DA supramolecular nanostructures^38,39^. Our longstanding interest^40–44^ in DA molecular assemblies and PDA supramolecules have led to observations that show that DA moieties can be readily introduced into target building blocks, and that morphological similarities exist between assembled DAs and irradiation-produced PDAs. These findings have enabled us to devise a, potentially general strategy for trapping kinetically favored metastable intermediates in self-assembly pathways, which utilizes PDA-forming photopolymerization. Specifically, we envisaged that the presence of a readily introduced DA moiety in a substance undergoing self-assembly would enable trapping of kinetically formed metastable intermediates through UV irradiation-induced formation of the morphologically stable PDA derivative. It should be noted that early studies on self-assembly of aldonamide and phosphatitylcholine-derived amphiphilic DAs revealed the existence of intriguing helical and tubular structures.^16–18^ In addition, UV-induced stabilization of the thermodynamically formed diacetylenic aldonamide nanotubes was reported earlier.^16^

**Fig. 1:**
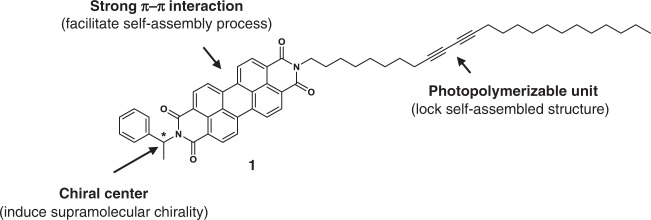
Molecular design. Molecular structure of diacetylene-containing chiral perylene diimide (PDI) **1** and roles played by the functional elements.

To explore the viability of the approach, self-assembly of the chiral PDI (**1**, Fig. 1), possessing a DA containing a long alkyl chain, was investigated. Owing to their intriguing optoelectronic properties, PDI derivatives have been subjected to extensive studies in various areas related to sensors, solar cells, and organic field effect transistors^45–51^. It was expected that DA-containing chiral **1** would undergo self-assembly to form well-defined supramolecules as a consequence of favorable *π*–*π* stacking and van der Waals interactions. In addition, it was anticipated that the presence of the chiral headgroup in **1** would induce chirality in supramolecular intermediates and the final product in the self-assembly pathway. Moreover, we reasoned that irradiation-promoted crosslinking of the DA moiety in **1** would cause the kinetic intermediates formed at different stages of the self-assembly process to be trapped by blocking their dissociation and morphological reorganization.

## Results

### Self-assembly

**Fig. 2:**
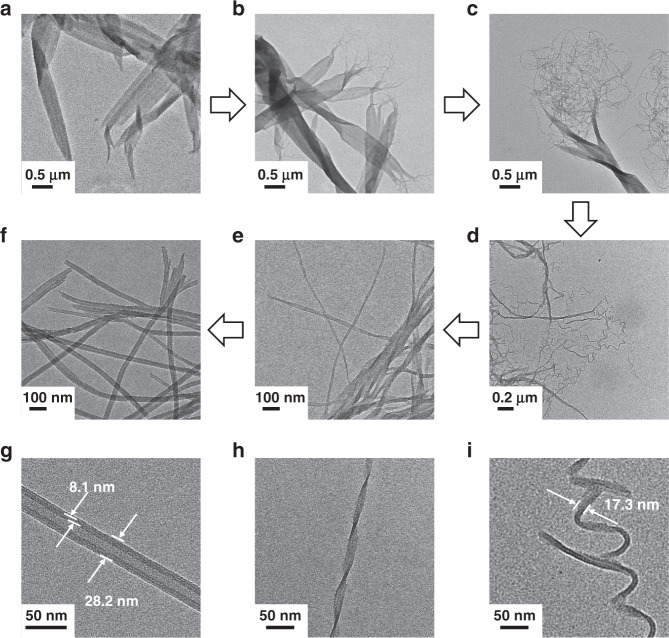
Time evolution of morphological nanostructures of **1-R** from nanoribbons to nanotubes. Transmission electron microscopy (TEM) images showing the transformation from nanoribbons to nanotubes during self-assembly of **1-R**. The initially formed nanoribbons (**a**) (5 min) begin to develop split ends (**b**) (10 min) and dissociate further (**c**) (30 min) to form nanocoils (**d**) (1 h), which are transformed to twisted nanofibers (**e**) (3 h), and eventually to nanotubes (**f**) (5 h). **g**–**i** TEM images of individual nanotube (**g**), nanohelix (**h**), and nanocoil (**i**). Self-assembly condition: 27 μM of **1-R** in 10% CHCl_3_/EtOH, 65 °C (clear solution) to 15 °C in a temperature-controlled cell, cooling rate: 10 °C/min.

To investigate the self-assembly process, a clear solution was prepared by mixing 0.3 mL of **1-R** (0.3 mM) (Supplementary Information Section 4) in chloroform and 3 mL of ethanol (**1-R** final concentration 27 μM) followed by heating the mixture to 65 °C. Cooling the hot solution of **1-R** to 15 °C in a temperature-controlled cell initiates a self-assembly process that results in rapid formation of metastable nanoribbons, which then slowly convert to nanotubes through several intermediate nanostructures (Fig. 2). At ~40 °C, metastable nanoribbons begin to form in the solution of **1-R** and are fully produced when the temperature is lowered to 15 °C. As displayed in Fig. 2a, the nanoribbons have a width of 300–500 nm and a length of several micrometers. Atomic force microscopy (AFM) analysis revealed that the self-assembled nanoribbons have an approximate height of 7 nm, which corresponds to that of a bilayer of **1-R** (Supplementary Fig. 5). Interestingly, ~10 min after reaching 15 °C, the nanoribbons begin to develop split ends (Fig. 2b) as a part of a time-dependent process (Fig. 2c–d after 0.5–1.0 h), which leads to their transformation to nanowires/nanocoils (Fig. 2i). A continuation of the self-assembly process for 3 h at 15 °C leads to formation of twisted nanohelices (Fig. 2e, h) and after 5 h nanotubes (Fig. 2f, g) are generated as the final thermodynamically most stable self-assembled structure of **1-R**. A similar morphological evolution of nanostructures is observed using the **1-S** antipode (Supplementary Fig. 6).

**Fig. 3:**
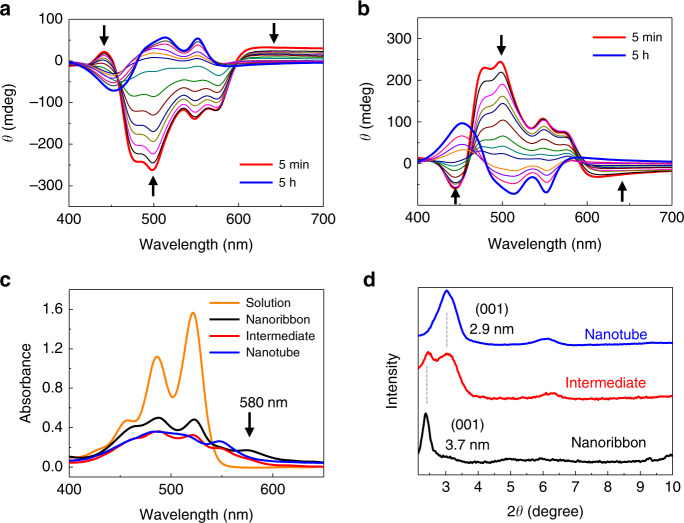
Spectroscopic monitoring of the self-assembly process. **a**, **b** Time-dependent changes in CD spectra of 10% CHCl_3_/EtOH solutions (27 μM) of **1-R** (**a**) and **1-S** (**b**) obtained by heating at 65 °C followed by cooling at 15 °C from 0 to 5 h. **c** Visible absorption spectra of **1-R** obtained at 65 °C (orange line, unassembled **1-R**), 5 min (black line), 3 h (red line), and 5 h (blue line) following initiation of self-assembly by cooling a heated solution from 65 to 15 °C. **d** Powder XRD spectra of **1-R** obtained 5 min (black line), 3 h (red line), and 5 h (blue line) following initiation of self-assembly by cooling a heated solution from 65 °C to 15 °C.

In addition to undergoing interesting morphological transitions, the DA-containing PDIs experience remarkable changes in absolute configurations at the supramolecular stereogenic centers during self-assembly^52,53^. This feature was probed by monitoring time-dependent circular dichroism (CD) spectra of 10% CHCl_3_/EtOH solutions (27 μM) of **1-R** and **1-S** (Fig. 3a, b). To examine the effect of linear dichroism (LD) on the CD signal, we monitored LD spectra during the self-assembly process. Only very weak LD signals were observed (Supplementary Fig. 7), confirming that the CD spectra observed with **1-R** is predominately a consequence of supramolecular chirality, generated from the self-assembly of the chiral building block. As displayed in the CD spectra, the initially formed metastable nanoribbons and thermodynamically stable nanotubes derived from each enantiomer display opposite supramolecular stereochemical configurations stemming from different stereochemical molecular stacking arrangements of the PDI units. In the case of **1-R**, the PDI units in the nanoribbons are stacked in a left-hand helical manner (negative Cotton effect), whereas those in nanotubes of **1-R** are stacked in a right-hand helical manner (positive Cotton effect). The difference in the stereogenic arrangements of PDI moieties in these morphologies was confirmed by using density functional theory (DFT) calculations (Supplementary Figs. 8 and 9). Thus, the transmission electron microscopy (TEM) images (Fig. 2 and Supplementary Fig. 6) and CD spectra (Fig. 3a, b) clearly demonstrate that the initially formed nanoribbon intermediates in the pathway for self-assembly of **1** slowly transform to thermodynamically more stable nanotubes with inversion of absolute configuration at their supramolecular stereogenic centers. It should be noted that, when a 1 : 1 mixture of **1-R** and **1-S** is used, the chirality inversion is not observed (Supplementary Fig. 10). As expected, the ribbon-to-tube transformation, observed with pure enantiomers **1-R** and **1-S**, does not occur with the racemic mixture. Thus, the initially formed nanoribbons are maintained even after 17 h (Supplementary Fig. 11).

In addition to CD spectroscopy, UV-visible absorption and X-ray diffraction (XRD) spectra were utilized to characterize morphological differences in self-assemblies of PDI **1** that arise from different molecular stacking arrangements. Analysis of UV-visible absorption spectra (Fig. 3c) shows that the intensity of the vibronic peak at 580 nm associated with nanoribbons decreases while that of the peak at 550 nm arising from nanotubes increases. The shape and peak position of UV-visible absorption spectra compared to those of unassembled PDI monomers indicate that **1-R** in the nanoribbons and nanotubes are stacked in the form of H-aggregates (twisted π-stacking) with left-handed and right-handed helical structures, respectively^54^. In addition, as J-type aggregates of PDI chromophores generally exhibit absorption at above 600 nm^45^, both nanoribbons and nanotubes are believed to be molecularly stacked in H-type forms. Hestand and Spano^55^ reported on the theoretically calculated UV-visible absorption spectra of twisted π-stacked PDI chromophores. Close inspection of the UV-visible absorption spectra of **1-R** in the nanoribbon and nanotube forms revealed that similarities exist between the observed and calculated spectra in terms of the shape and peak position. Although it is difficult to specify the dihedral angle between twisted π-stacked PDI chromophores, it is expected that reduction of the dihedral angle occurs when the nanoribbons are transformed into nanotubes. Furthermore, UV-induced polymerization occurs more favorably with the nanotubes than the nanoribbons. Thus, it is reasonable to speculate that the DA-containing alkyl chains are aligned more favorably with reduced dihedral angles for the topochemical polymerization in the nanotubes.

Examination of the XRD spectra displayed in Fig. 3d shows that structural differences exist between nanoribbons and nanotubes. The peak at 2*θ* = 2.38° in the XRD spectrum of the nanoribbons corresponds to a *d*-spacing of 3.7 nm, whereas the peak at 2*θ* = 3.04° in that of the nanotubes is associated with a *d*-spacing of 2.9 nm.

### Light-promoted trapping of metastable intermediates

**Fig. 4:**
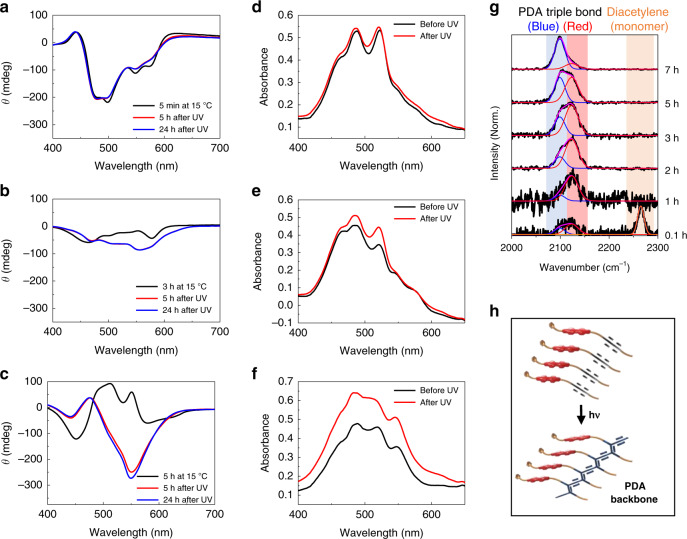
Light-promoted trapping of metastable intermediates formed during self-assembly. **a**–**c** CD spectra of 10% CHCl_3_/EtOH solutions (27 μM) of **1-R** obtained by heating at 65 °C followed by cooling at 15 °C for 5 min (**a**), 3 h (**b**), and 5 h (**c**) (black line), respectively. Red and blue lines represent the CD spectra obtained after UV light irradiation (254 nm, 25 mW m^−2^, 10 s) followed by standing at 15 °C for 5 h (red line) and 24 h (blue line). **d**–**f** Black lines represents the UV-visible absorption spectra of 10% CHCl_3_/EtOH solutions (27 μM) of **1-R** obtained by heating at 65 °C followed by cooling at 15 °C for 5 min (**d**), 3 h (**e**), and 5 h (**f**), respectively. Red lines represent the UV-visible absorption spectra obtained after UV light irradiation (254 nm, 25 mW cm^−2^, 10 s) of the same solutions. **g** Raman spectra obtained after UV light irradiation (254 nm, 25 mW cm^−2^, 10 s) of **1-R** at different self-assembly time scale at 15 °C. Monomers in solution was removed by centrifugation to monitor PDA formation in the self-assembled supramolecules. **h** Schematic of a light-induced PDA formation from self-assembled DA monomers.

To assess the feasibility of the light-promoted trapping of intermediates, solutions of **1-R** at different stages in the self-assembly process were irradiated with UV light (Fig. 4a–c). The CD spectra of a 10% CHCl_3_/EtOH solution (27 μM) of **1-R** obtained by heating the solution to 65 °C followed by cooling to 15 °C for 5 min is displayed in Fig. 4a (black line). The red and blue lines in Fig. 4a represent CD spectra of UV-irradiated (254 nm, 25 mW cm^−2^, 10 s) nanoribbon solution after standing for 5 h (red line) and 24 h (blue line) at 15 °C, respectively. Interestingly, CD monitoring of the process shows that chirality inversion associated with the nanoribbon to nanotube morphological change of **1-R** is completely blocked by UV irradiation. Similar light-promoted blocking of the chirality inversion was also observed using the **1-S** antipode (Supplementary Fig. 12). In addition, the CD signal corresponding to the metastable nanoribbon structure of **1-R** is maintained even after 24 h at 15 °C following UV irradiation. Also, inspection of TEM images of UV-irradiated solutions of **1-R** and **1-S** after 24 h at 15 °C reveals the existence of nanoribbon structures (Supplementary Fig. 13). Finally, the exceptional stabilities of the photopolymerization-trapped, kinetically preferred nanoribbon intermediates in the pathways for self-assembly of **1-R** and **1-S** are further evidenced by the observation that their morphology is maintained even after being kept in solution for 3–5 months at room temperature (Supplementary Fig. 14).

The UV-induced termination of self-assembly of PDI **1** at the early nanoribbon stage is a consequence of formation of conjugated ene-yne backbone structures, which induces crosslinking of the DA units that effectively prevents dissociation of the self-assembled structure (see below)^32–37^. The results of further studies showed that UV-induced termination of self-assembly can be accomplished at later stages of self-assembly. Specifically, UV irradiation of a solution of **1-R** 3 h after initiation of self-assembly at 15 °C causes termination of the CD inversion (Fig. 4b). TEM images of the UV-irradiated solution show that a mixture of nanocoils and helical nanowires exist (Supplementary Fig. 15). It should be noted that the CD wavelength change occurring after UV irradiation is due to the presence of the PDA chromophore (see below). The UV irradiation trapping protocol was also performed at the last stage of self-assembly. As displayed in Fig. 4c (red line), UV irradiation of the tubular solution of **1-R** results in generation of a strong negative Cotton effect owing to formation of the macroscopic chiral PDA backbone. The intensity and shape of the CD spectrum remains even after 24 h (Fig. 4c, blue line) and UV irradiation does not lead to alteration of the morphology, because only tubular structures are observed (Supplementary Fig. 16).

To gain more information about the UV-induced formation of the PDA backbone, UV-visible absorption and Raman spectroscopic analyses were carried out. As displayed in Fig. 4d–f, UV irradiation of independent **1-R** solutions containing nanoribbons (Fig. 4d), nanowires (Fig. 4e), and nanotubes (Fig. 4f) promotes increases in the intensities of bands in the visible region. In addition, the intensity change is more profound for the nanowire and nanotube solutions, indicating the PDA formation is more efficient at the later stage of self-assembly. Additional evidence suggesting that PDA formation promoted by UV irradiation is more effective at the later stage of self-assembly was obtained by using Raman spectroscopy. As shown in Fig. 4g, Raman spectra of nanoribbons of **1-R** following UV irradiation (254 nm, 25 mW cm^−2^, 10 s) contain a descending peak at 2263 cm^−1^ associated with unpolymerized DA and an ascending peak at 2124 cm^−1^ associated with the PDA ene-yne backbone^56^. The weak polymer peak suggests that a relatively short PDA backbone is generated by UV irradiation of the kinetically preferred metastable nanoribbon self-assembled intermediate. This is likely a consequence of the low packing tightness and/or poor spatial arrangement of the DA moieties in nanoribbons of **1-R**. It is evident that the monomer peak disappears and the polymer peak dominates at the later stage, indicating longer PDA chains are produced by UV irradiation. Interestingly, close inspection of the polymer peak suggests that PDAs in blue phase are preferentially produced at the later stage, confirming that the DAs are more effectively stacked for the topochemical polymerization at the later stage. Figure 4h shows the structural transformation of a self-assembled DA supramolecule to a PDA.

To investigate the effect of UV irradiation time on the self-assembly process^57^, CD spectra and TEM images were recorded following various irradiation times. Interestingly, short time (1.5, 3, or 5 s) UV irradiation of the **1-R** nanoribbon solution resulted in partial disassembly of the nanoribbons (Supplementary Fig. 17). As evidenced by the CD spectra, the chirality inversion process proceeds to a certain degree and eventually stops. In addition, monitoring of TEM images confirmed that metastable nanoribbons were partially transformed into nanowires using short irradiation times. These results indicate that 10 s irradiation, which is used in this study, is required for efficient trapping of the metastable intermediates. Interestingly, the results suggest that the self-assembly process can be controlled by manipulating the UV irradiation time, another potentially meritorious feature of the light-directed method.

UV irradiation was found to not only block the self-assembly process but also to afford stabilization of the metastable intermediates, owing to covalent modification of the supramolecules. For instance, inspection of TEM images shows that the nanoribbon structures are maintained 24 h after a **1-R** ribbon solution is UV-irradiated and heat-treated (65 °C, 10 min) (Supplementary Fig. 18). The UV-promoted enhanced stabilization of the supramolecules also occurs at the last stage. To demonstrate this, a solution of the nanotubular morphological form of **1-R**, produced by self-assembly at 15 °C for 5 h (see Fig. 3a), was heated to 65 °C. This operation results in the disappearance of the CD signal (Supplementary Fig. 19, black line), which indicates that the self-assembled nanotubes dissociate into individual PDI molecules because **1-R** in solution does not display a CD signal (Supplementary Fig. 20). As monitored by using CD spectroscopy, cooling this solution to 23 °C (Supplementary Fig. 19, red line) and then 15 °C (Supplementary Fig. 19, blue line) results in reformation of **1-R** nanoribbons. The nanoribbons were found to convert slowly to nanotubes at 15 °C with inversion of supramolecular chirality (Supplementary Fig. 21). In sharp contrast, heat treatment (65 °C) of a UV-irradiated (254 nm, 25 mW cm^−2^, 10 s) solution of nanotubular **1-R** results in no morphological changes and thus tubular structures are preserved (Supplementary Fig. 22) and supramolecular chirality is retained (Supplementary Fig. 23).

The efficient photoinduced trapping of kinetic intermediates enables a comparison of the photocurrents of the nanoribbon and nanotubes. The thermodynamically stable nanotubes display an increased photocurrent compared to the nanoribbon intermediates (Supplementary Fig. 24a). This result is directly reflected in the gas sensing property of the nanostructures. It is well known that photocurrents of self-assembled PDIs increase upon exposure to gaseous NH_3_ owing to the operation of a photoinduced electron transfer mechanism (NH_3_ donor and PDI acceptor). Moreover, it has been demonstrated that the photocurrents of PDI aggregates increase as the overlap between the PDI moieties increase^28^. In order to assess the gas sensing properties of the self-assembled structures, photocurrents of thin films of UV-photopolymerized forms of **1-R** nanoribbons and nanotubes were measured before and after exposure to different concentrations of NH_3_. Inspection of the plot given in Supplementary Fig. 24b shows that the UV-irradiated nanotube displays a modestly large photocurrent, and that the magnitude of the current is enhanced upon addition of NH_3_ gas. In contrast, **1-R** nanoribbons do not display an observable photocurrent either in the absence or presence of NH_3_. The strikingly different photocurrents and responses to NH_3_ between the two morphologically different forms of **1-R** indicate that the PDI chromophores are relatively loosely packed in the nanoribbon form.

## Discussion

**Fig. 5:**
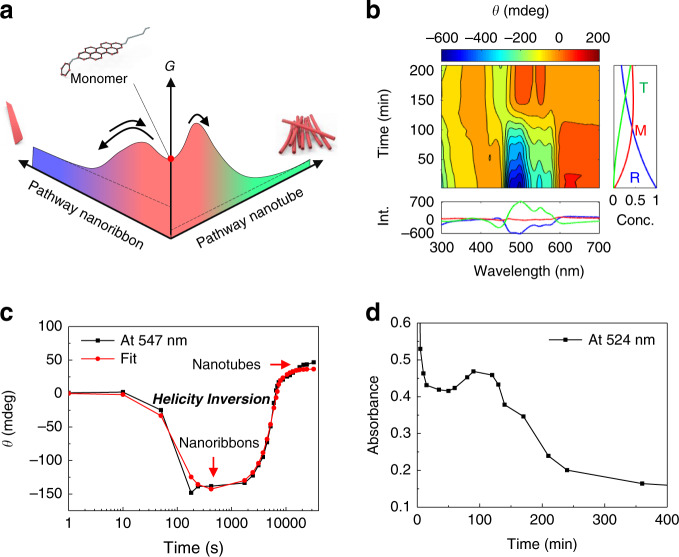
Kinetics of the self-assembly process. **a** Reaction profile for the self-assembly process. **b** Results of global fitting analysis based on the kinetic model in Supplementary Fig. 24a of time-dependent CD spectra of **1-R**. The CD spectra used to determine the time-dependent population evolution/decay (right panel) of nanoribbon (R, blue), monomer (M, red), and nanotube (T, green) are shown in the bottom panel. **c** Time dependence of CD signal intensities at 547 nm following initiation of self-assembly by cooling a heated solution from 65 to 15 °C. **d** Time-dependent absorbance (at 524 nm) of PDI monomers in solution after removing self-assembled supramolecules following initiation of self-assembly by cooling a heated solution from 65 °C to 15 °C.

The pathway for self-assembly of PDI **1** can be described by using either an isodesmic or cooperative (nucleation-elongation) model in a manner to that shown by Meijer and colleagues^21–23^. In the isodesmic model, the self-assembly process is represented by a single equilibrium constant, *K*_iso_ = *k*_asso_/*k*_disso_ where *k*_asso_ and *k*_disso_ are respective association and dissociation rate constants. In contrast, in the cooperative model, self-assembly is characterized by two equilibrium constants, *K*_n_ and *K*_e_, which correspond to nucleation and elongation steps, respectively, and where normally *K*_n_ < *K*_e_. The self-assembly kinetics of the PDI derivative probed in the current study, schematically depicted in the graph shown in Fig. 5a, correspond to the situation in which cooling the solution leads to rapid formation of metastable nanoribbons, which then gradually form thermodynamically more stable nanotubes. As a lag phase is present in the concentration-dependent self-assembly process, the formation of nanoribbons can be properly described by using the cooperative model (Supplementary Information Section 8 and Supplementary Fig. 25)^21^^–^^23^. In addition, slow nanotube formation is best described by using the cooperative model as well. By analyzing the time dependence of the changes taking place in the CD signal at 547 nm (Fig. 5c) using the two competing cooperative models and the previously described method^21–23^, we determined that the equilibrium constants for the respective nucleation and elongation steps for nanoribbon **1-R** formation at 15 °C are *K*_n_ = 6.96 × 10^2^ M^−1^ and *K*_e_ = 3.20 × 10^4^ M^−1^, and for nanotube formation are *K*_n_ = 1.19 × 10^3^ M^−1^ and *K*_e_ = 6.39 × 10^4^ M^−1^.

The nanoribbon-to-nanotube conversion process in solution was also analyzed by using a global fitting analysis based on the kinetic model given in Supplementary Fig. 26a. The results of analysis of the time-dependent changes in the CD signal of **1-R** are summarized in the plot in Fig. 5b and Supplementary Fig. 27^58,59^. The observations demonstrate that the evolution/decay times of the spectral features associated with the nanoribbons and nanotubes produced by self-assembly of **1-R** are well resolved. In addition, analysis of UV-visible absorption spectra of solutions from which self-assembled supramolecules are removed shows that the PDI monomer concentration increases (*k*_1_ = 121 min) when nanoribbons in solutions disappear gradually, and then it slowly decreases as nanotubes are produced (*k*_2_ = 191 min). These observations clearly demonstrate that conversion of nanoribbons to nanotubes in solution occurs by dissociation of the nanoribbons into PDI monomers, followed by subsequent reassembly of the PDI monomers to form nanotubes. The transformation of nanoribbons into nanotubes can proceed via either a consecutive or competitive pathway. The observation of the transient increase in the concentration of PDI monomer (Fig. 5d) suggests that the self-assembly process should occur via a competitive pathway. Concentration-dependent kinetic studies were carried out to clearly distinguish between consecutive and competitive pathways. If the transformation of nanoribbons into nanotubes is accelerated by increasing the concentration of monomers, then a consecutive pathway is involved. On the other hand, if the lag time increases with increasing the concentration, a competitive pathway is responsible for the transformation^24^. The rates at which nanoribbons are transformed into nanotubes were determined using different concentrations of **1-R** by measuring the CD spectra as a function of time. To compare the transformation rates, the degree of aggregation (*α*) was plotted as a function of time at a given concentration (Supplementary Fig. 28). The results show that the transformation of nanoribbons into nanotubes slows as the concentration increases, demonstrating that the transformation process occurs through a competitive pathway.

**Fig. 6:**
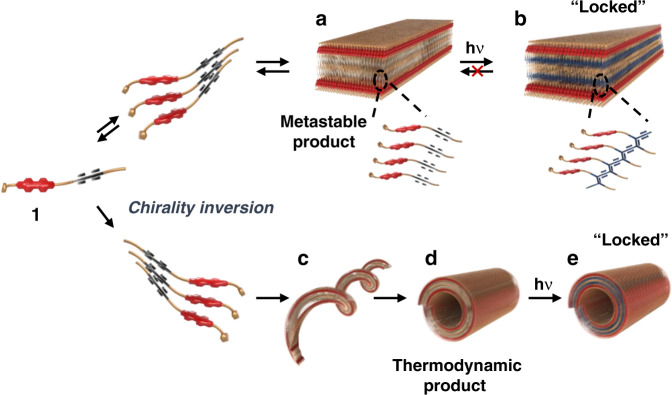
Pathway for self-assembly of 1. A kinetically favored metastable nanoribbon is generated in an initial nucleation step (**a**). UV irradiation of this kinetic intermediate promotes polymerization and prevents dissasembly of the nanorribbon (**b**). Without UV irradiation, the initially generated nanoribbon dissociates to form monomers, which reassemble to yield a helical intermediate (**c**), which is slowly transformed into the thermodynamically stable nanotube with inversion of the supramolecular chirality (**d**). Polymerization of diacetylene units in the nanotube by using UV light irradiation affords covalent modification of the tube (**e**).

The overall schematic representation of the self-assembly pathways for PDI **1** is depicted in Fig. 6. In the route, self-assembly of **1** takes place via an initial nucleation step that generates a kinetically preferred metastable nanoribbon (Fig. 6a). UV irradiation-promoted polymerization of the DA moiety in the nanoribbon traps this kinetically preferred intermediate by preventing it from disassembling and eventually dissociating to reform monomers (Fig. 6b). In the absence of trapping, the kinetically formed nanoribbon dissociates to reform monomers, which more slowly reassemble to generate a helical intermediate (Fig. 6c), which converts into the thermodynamically stable nanotube (Fig. 6d). Like it does in the case of the nanoribbon, UV-irradiation creates a covalently trapped nanotube (Fig. 6e), which maintains its morphological integrity during thermal cycles. Formation of nanotubes from helical intermediates was further evidenced by observing that helical structures form when polymerized nanotubes are incubated in organic solvents (Supplementary Fig. 29).

In summary, we developed an efficient photopolymerization based strategy for “trapping” metastable intermediates in self-assembly processes. The presence of the photopolymerizable DA moiety in the PDI enables the kinetically favored nanoribbon intermediate formed early in the self-assembly process to be effectively trapped by using UV irradiation. Importantly, the trapped nanoribbon retains its structure and supramolecular chirality in the assembly medium over a several month period. In addition, we demonstrated that the photoinduced trapping strategy could be applied to the later stage of the self-assembly process, enabling capture of nanocoil/nanohelices intermediates by using UV irradiation. Furthermore, UV irradiation of the nanotube formed at the last stage in the self-assembly pathway leads to generation of a rigid nanotube that is stable under heating and solvent processing. In light of the technical difficulty associated with current methods for capturing kinetic intermediates in the solution state, the strategy developed in this study should be an important contribution to this field.

## Methods

Details of the synthesis of the enantiomers of PDI **1**, characterization data and sample preparation for self-assembly, as well as spectroscopic, microscopic, and computational investigations are provided in Supplementary Information.

## Supplementary information

Supplementary Information

## Data Availability

The data that support the finding of this study are available in figshare with identifier [doi: 10.6084/m9.figshare.13176893]. These data are featured in Figs. 3, 4, and 5. All other relevant data supporting the key finding of this study are available within the article and its Supplementary Information files or from the corresponding author upon reasonable request.
