# Supplementary material for: Light-directed trapping of metastable intermediates in a self-assembly process
Source: Nat Commun. 2020 Dec 7;11:6260. doi: 10.1038/s41467-020-20172-6 (PMC7721704; doi:10.1038/s41467-020-20172-6)
Supplement: Supplementary file 1 — Supplementary Information [file 41467_2020_20172_MOESM1_ESM.pdf]

Supplementary Information

Light-directed trapping of metastable intermediates in a self-assembly process

Joonsik Seo,[1,†] Joonyoung F. Joung,[2,†] Sungnam Park,[2,*] Young Ji Son,[3] Jaegeun Noh,[3,4] and Jong-Man Kim[1,4]*

[1]Department of Chemical Engineering, Hanyang University, Seoul 04763, Korea

[2]Department of Chemistry and Research Institute for Natural Science, Korea University, Seoul, 02841, Korea

[3]Department of Chemistry, Hanyang University, Seoul 04763, Korea

[4]Institute of Nano Science and Technology, Hanyang University, Seoul 04763, Korea

*E-mail: spark8@korea.ac.kr (S.P.), jmk@hanyang.ac.kr (J.-M. Kim)

Contents **Page no.**

1. Instruments……………………………………………………………..........2

2. Materials……………………………………………………………….........2

3. Synthesis of chiral PDI 1-R and 1-S..3

4. Self-assembly of chiral PDI 1 and light controlled trapping.............................4

5. Photocurrent measurement...4

6. Quantum chemical calculations...4

7. Global fitting analysis…………………………………………………........5-6

8. Cooperative model describing self-assembly of 1-R ………………………........7

9. Table S1..8

10. Supplementary Figures 2-29...9-36

11. References...37

1. Instruments

TEM images were obtained using JEOL JEM-2010 and JEM-2100F microscope. Raman spectra were recorded on a LabRAM HR Evlution Raman spectrometer (Horiba Scientific, λ_{ex}=785 nm). UV-vis absorption spectra were recorded on a single beam Agilent 8453 UV−vis spectrometer (Agilent Technologies, Waldbronn, Germany). CD spectra were recorded on J-1500 CD spectrometer (Jasco). ^{1}H NMR and ^{13}C NMR were recorded on a Varian Unitylnova (300 MHz, 75 MHz) at 298 K in $CDCl_3$. Mass spectra (Matrix assisted laser desorption ionization time-of-flight mass spectroscopy, MALDI-TOF) was recorded on an AXMA (Shimadzu). XRD spectra were recorded using a minFlex600. Direct-current conductivity measurements of the fibers were performed using a two-line-probe method with a Keithley 237 source-measure unit.

2. Materials

10,12-pentacosadiynoic acid (2) was purchased from GFS chemical (Powell, OH). The intermediates 4[1], 7[2] and 9[3] were prepared according to the known procedures. Perylene-3,4,9,10-tetracarboxylic dianhydride, dichloromethane (DCM) and diphenyl phosphoryl azide (DPPA) were purchased from Sigma-Aldrich (Korea). Trifluoroacetic acid (TFA), (R)-(+)-phenylethylamine and (S)-(-)-phenylethylamine were purchased from Tokyo Chemical Industry (Korea).

3. Synthesis of chiral PDI 1-R and 1-S

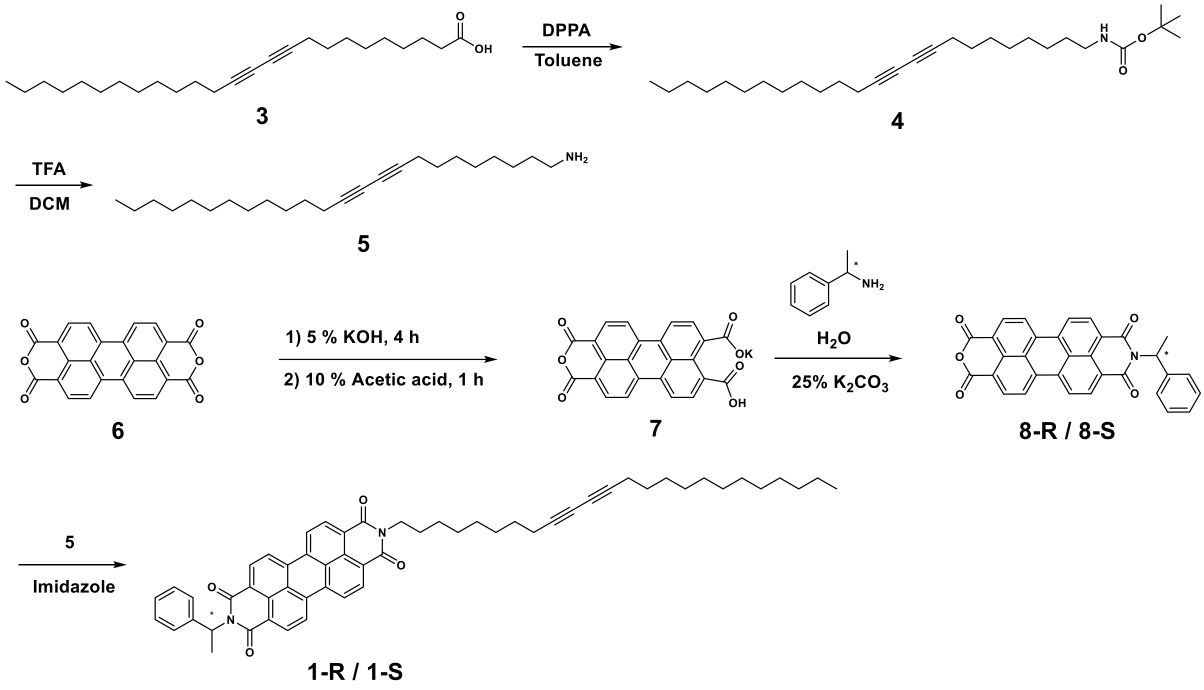

Supplementary Figure 1. Synthesis of diacetylene-containing chiral PDI 1-R and 1-S.

Synthesis of 1-R and 1-S. A mixture of 5 (0.29 g, 0.8 mmol) and 8-R (0.2 g, 0.4 mmol) in imidazole (3 g) was stirred at 100 °C for 8 h. After cooling to room temperature, ethanol and concentrated HCl (12 N) were added to the mixture and stirred for overnight. The resulting red solid was collected by vacuum filtration through a 0.45 μm membrane filter and rinsed thoroughly with water and ethanol. The residue was purified by a silica gel column chromatography (chloroform : acetone = 100 : 1) to afford 1-R (0.18 g, 55 %) as a red solid. m.p: 275 °C (decompose); ^{1}H NMR (300 MHz, $CDCl_3$) : δ 8.47-8.52 (m, 4 H), 8.31-8.35 (m, 4 H), 7.58 (d, J = 7.5 Hz, 2 H), 7.37 (t, J = 7.2 Hz, 2 H), 7.26-7.30 (m, 1 H), 6.56 (q, J = 7.2 Hz, 1H), 4.15 (t, J = 6.9 Hz, 2 H), 2.21-2.26 (m, 4 H), 2.07 (d, J = 7.2 Hz, 3 H), 1.75 (m, 2 H), 1.25-1.56 (m, 32 H), 0.87 (t, J = 6.6 Hz, 3 H). ^{13}C NMR (75 MHz, $CDCl_3$) : δ 163.2, 163.1, 140.5, 134.2, 131.3, 131.0, 129.2, 129.0, 128.2, 127.4, 127.1, 126.1, 126.0, 123.5, 123.1, 122.9, 122.8, 77.6, 77.4, 77.2, 77.0, 76.6, 65.3, 65.2, 50.5, 40.6, 31.9, 29.6, 29.5, 29.3, 29.2, 29.1, 29.0,

28.8, 28.3, 28.3, 28.0, 27.1, 22.7, 19.2, 16.3, 14.1. MS (MALDI-TOF, *m/z*) : exact mass calculated for $C_{56}H_{58}N_2O_4$ required 823.44 found 823.04.

By employing a similar protocol, the enantiomeric counterpart 1-S was prepared (yield: 48%).

4. Self-assembly of chiral PDI 1 and light controlled trapping

A clear solution of the chiral PDI 1-R or 1-S was prepared by mixing 0.3 mL of 1-R or 1-S (0.3 mM) in chloroform with 3 mL of ethanol (final concentration: 27 μM) followed by heating the mixture to 65 ℃. Cooling (cooling rate: 10 ºC/min) the hot solution of 1-R or 1-S to 15 ºC in a temperature controlled cell initiated self-assembly process, resulting in fast formation of metastable nanoribbons which were slowly converted into nanotubes through intermediate nanostructures. Various spectroscopic (UV-visible, fluorescence, circular dichroism) and microscopic (TEM and AFM) methods were employed to analyze the kinetic and thermodynamic products. Light controlled experiments were carried out by irradiation of 254 nm UV light (25 mW cm^{-2}, 10 sec) to the self-assembling solution at different stages of the self-assembly process.

5. Photocurrent measurement

Photocurrent measurements were carried out using a two-probe method with a Keithley 237 source-measure unit in a shielded dark box to avoid unwanted electromagnetic radiation. To fabricate sample electrode, a 100 μL (27 μM) of UV-irradiated (254 nm, 25 mW cm^{-2}, 10 sec) 1-R aggregates solutions (nanoribbons and nanotubes) were dropped on interdigitated electrodes (ED-IDE1-Au, MICRUX TECHNOLOGIES) for electrical measurements. A halogen lamp (OSRAM, 12 V, 100 W) was used as a light source for photocurrent measurements.

6. Quantum chemical calculations

Aggregates of 1-R (and 1-S) is a huge supramolecular system and the quantum chemical calculations of aggregates is not practically feasible. Thus, the model molecules of 1-R and 1-S in Supplementary Figs. 8a and 9a were used for quantum chemical calculations. The model molecule of 1-R consists of perylenediimide (PDI) molecule with 1-phenylethyl group as one

side and methyl group as another side. The alkyl chain with diacetylene group is truncated because the optical properties will belong to the chromophore, PDI group. All calculations were performed using the density functional theory (DFT) and time-dependent DFT methods (ωB97XD/6-31+G(d))[4,5] as implemented in the Gaussian 16 package.[6]

Optimized molecular structures of dimers were shown in Supplementary Figs. 8 and 9. Two left-handed and two right-handed dimers of 1-R and 1-S were optimized and their UV-visible absorption and circular dichroism (CD) spectra were calculated. For dimers of 1-R, left-handed and right-handed dimers exhibit negative and positive Cotton effects in CD spectra, respectively. On the contrary, left-handed and right-handed dimers of 1-S have positive and negative Cotton effects in CD spectra as shown in Supplementary Fig. 9d. Based on results of the DFT calculations, 1-R is initially aggregated to the left-handed self-assembly (nanoribbon) and then subsequently changed to the right-handed self-assembly (nanotube).

7. Global fitting analysis

The CD signals in Figure 3a contain all dynamic information on the self-assembly of 1-R. Here, we used the global fitting analysis to extract all dynamical information from the CD signals. In the global fitting analysis, the CD signal (**D**) can be expressed by the product of the time-dependent populations (**C**) and their corresponding spectral components ($\mathbf{S}^{\mathrm{T}}$),

$$\mathbf{D} = \mathbf{C} \cdot \mathbf{S}^{\mathrm{T}} \tag{1}$$

where the columns of matrix (**C**, sized $m \times k$) are the time-dependent populations of the kth components at the time delay of m and the rows of matrix ($\mathbf{S}^{\mathrm{T}}$, sized $k \times n$) are the corresponding k^{th} spectra as a function of wavelength n. And m, k, and n represent the time, the number of components, and the spectra, respectively. The detailed procedure of the global fitting analysis by using Matlab codes has been reported elsewhere.[7-11] Briefly, the coupled differential equations for the time-dependent populations of individual species in Figure 5a and Supplementary Fig. 26a are given by

$$\begin{aligned} \frac{d[\mathrm{R}]}{dt} &= -k_1[\mathrm{R}] \\ \frac{d[\mathrm{M}]}{dt} &= k_1[\mathrm{R}] - k_2[\mathrm{M}] \\ \frac{d[\mathrm{T}]}{dt} &= k_2[\mathrm{M}] \end{aligned} \tag{2}$$

where [R], [M], and [T] are the concentration of nanoribbon, monomer, and nanotube, respectively.

The analytical solutions are obtained by using $[\mathrm{R}]_0=1$,

$$
\begin{aligned}
[\mathrm{R}] &= \exp(-k_1 t) \\
[\mathrm{M}] &= \frac{k_1 \exp(-k_2 t)}{k_1 - k_2} - \frac{k_1 \exp(-k_1 t)}{k_1 - k_2} \\
[\mathrm{T}] &= \frac{k_2 \exp(-k_1 t)}{k_1 - k_2} - \frac{k_1 \exp(-k_2 t)}{k_1 - k_2} + 1
\end{aligned}
\tag{3}
$$

The CD signals are written as the sum of the individual components with their spectra and time-dependent populations. For a given kinetic model, the time-dependent populations (**C**) are calculated with the initial rate constants. The corresponding spectral components ($\mathbf{S}^{\mathrm{T}}$) are obtained by the product of the pseudo-inverse matrix of **C** and **D**,

$$\mathbf{S}^{\mathrm{T}} = \mathbf{C}^{-1} \cdot \mathbf{D} \tag{4}$$

The fitted CD signal ($\mathbf{D}^{\mathrm{fitted}}$) is constructed by the product of **C** and $\mathbf{S}^{\mathrm{T}}$,

$$\mathbf{D}^{\mathrm{fitted}} = \mathbf{C} \cdot \mathbf{S}^{\mathrm{T}} \tag{5}$$

The best fitted data in Supplementary Fig. 27 were obtained by iteratively varying the rate constants until the difference between the experimental data ($\mathbf{D}^{\mathrm{exp}}$) and the fitted data ($\mathbf{D}^{\mathrm{fitted}}$) was minimized.[7-9]

8. Cooperative model describing self-assembly of 1-R

The CD signals at 547 nm in Figure 5c are analysed based on the cooperative model proposed by Meijer.[11,12]

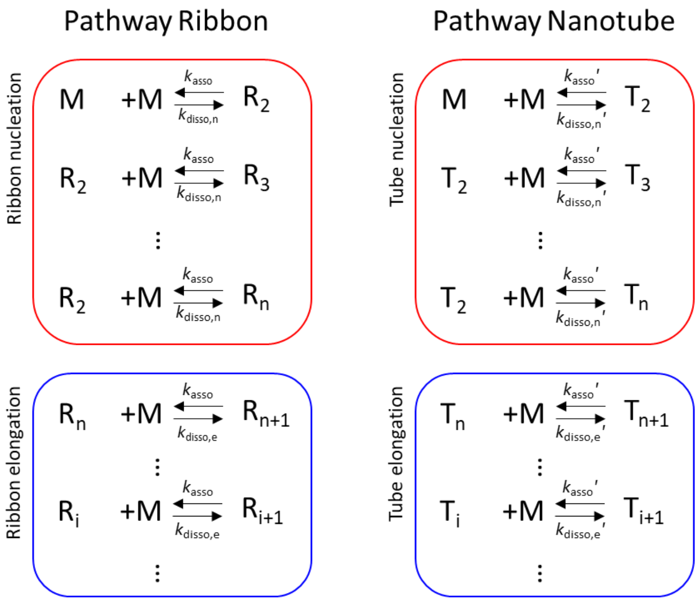

In cooperative model, the aggregates change its chain size in two parts. In the nucleation part, oligomers can change chain length upon monomer association and dissociation, with rate constants k_{asso} and $k_{disso,n}$, respectively. In the elongation part beyond the nucleus with chain length n, further elongation takes place with monomer association rate constant k_{asso}, and monomer dissociation is described with rate constant $k_{disso,e}$. The equilibrium constant for nucleation ($K_n = k_{asso} / k_{disso,n}$) is smaller than that for elongation ($K_e = k_{asso} / k_{disso,e}$) by definition of cooperative model. In this study, two cooperative models are competing to form nanoribbon (R) or nanotube (T). To fit the CD signals in Figure 4c, a maximum chain length is fixed to 50 and the nucleus sizes (n) for R and T are fixed to 5. The rate constants are summarized in Table S1. the equilibrium constants are found to be $K_{R,n} = 6.96\times10^2$ M^{-1}, $K_{R,e} = 3.20\times10^4$ M^{-1}, $K_{T,n} = 1.19\times10^3$ M^{-1}, and $K_{T,e} = 6.39\times10^4$ M^{-1} at 15 °C. The nanoribbon is the kinetically favored pathway and its association rate constant ($k_{asso} = 8.72\times10^5$ M^{-1} s^{-1}) is faster than association rate constant for nanotube ($k_{asso}' = 6.54\times10^3$ M^{-1} s^{-1}). But $K_{T,n}$ and $K_{T,e}$ are larger than $K_{R,n}$ and $K_{R,e}$, respectively, and thus the nanotube is the thermodynamically stable product.

9. Table S1. The rate constants for two competing cooperative models.

Pathway Nanoribbon		Pathway Nanotube	
k_{asso}	8.72×10^{5} M^{-1} s^{-1}	k_{asso}'	6.54×10^{3} M^{-1} s^{-1}
$k_{disso,n}$	1.25×10^{3} s^{-1}	$k_{disso,n}'$	5.49×10^{0} s^{-1}
$k_{disso,e}$	2.37×10^{1} s^{-1}	$k_{disso,e}'$	1.02×10^{-1} s^{-1}

10. Supplementary Figures 2-29

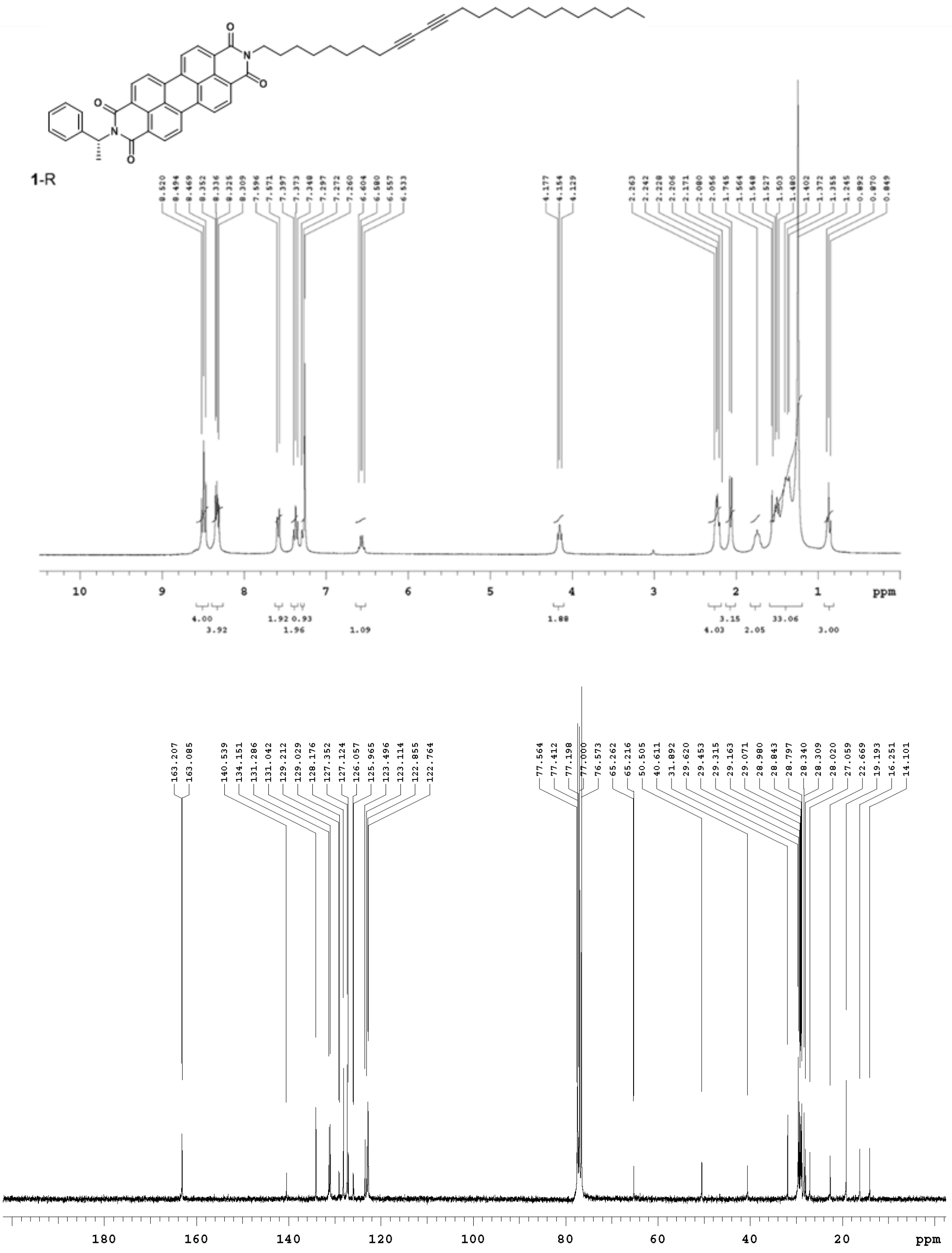

Supplementary Figure 2. ^{1}H NMR (top, 300 MHz, $CDCl_3$) and ^{13}C NMR (bottom, 75 MHz, $CDCl_3$) spectra of 1-R.

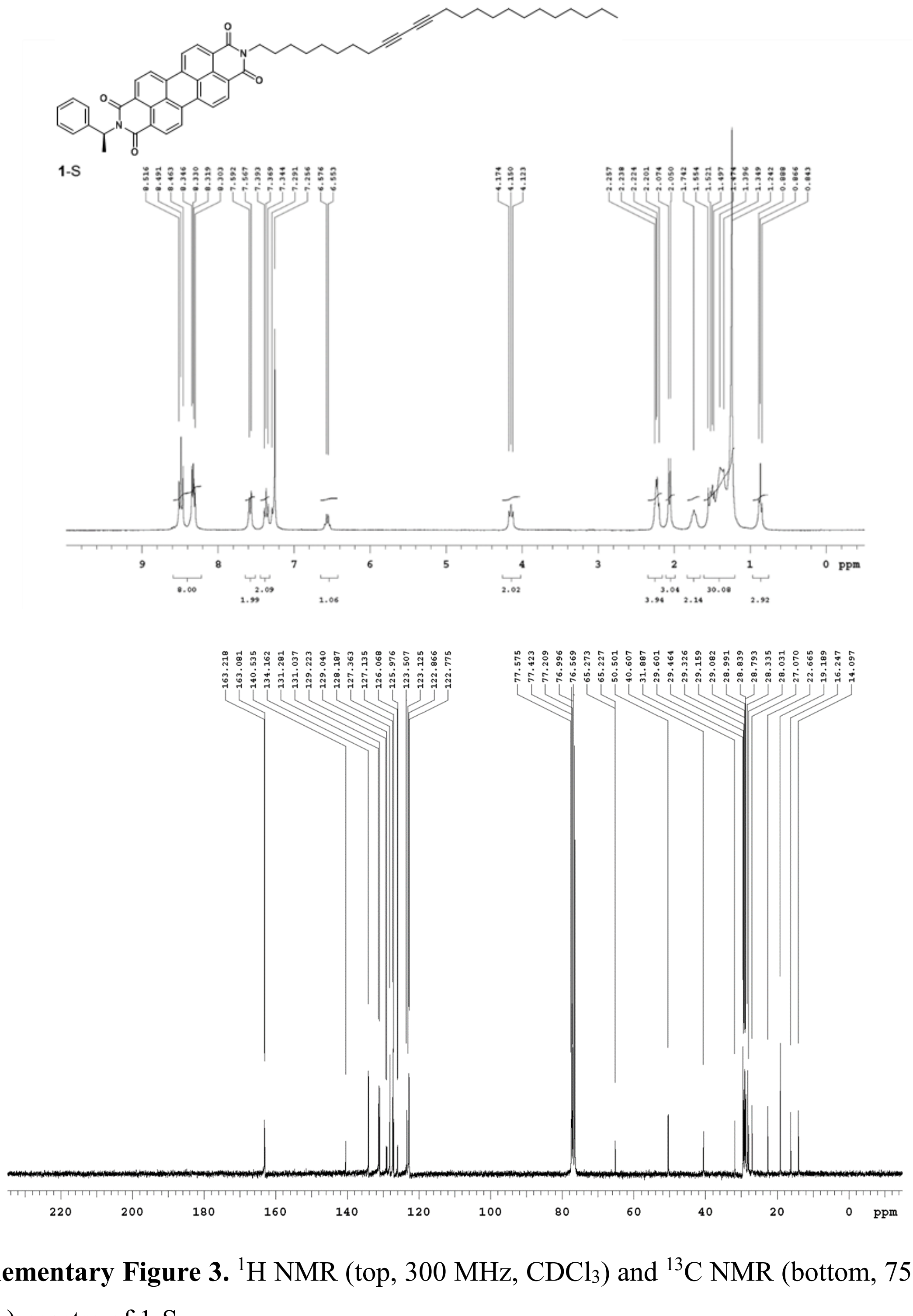

Supplementary Figure 3. ^{1}H NMR (top, 300 MHz, $CDCl_3$) and ^{13}C NMR (bottom, 75 MHz, $CDCl_3$) spectra of 1-S.

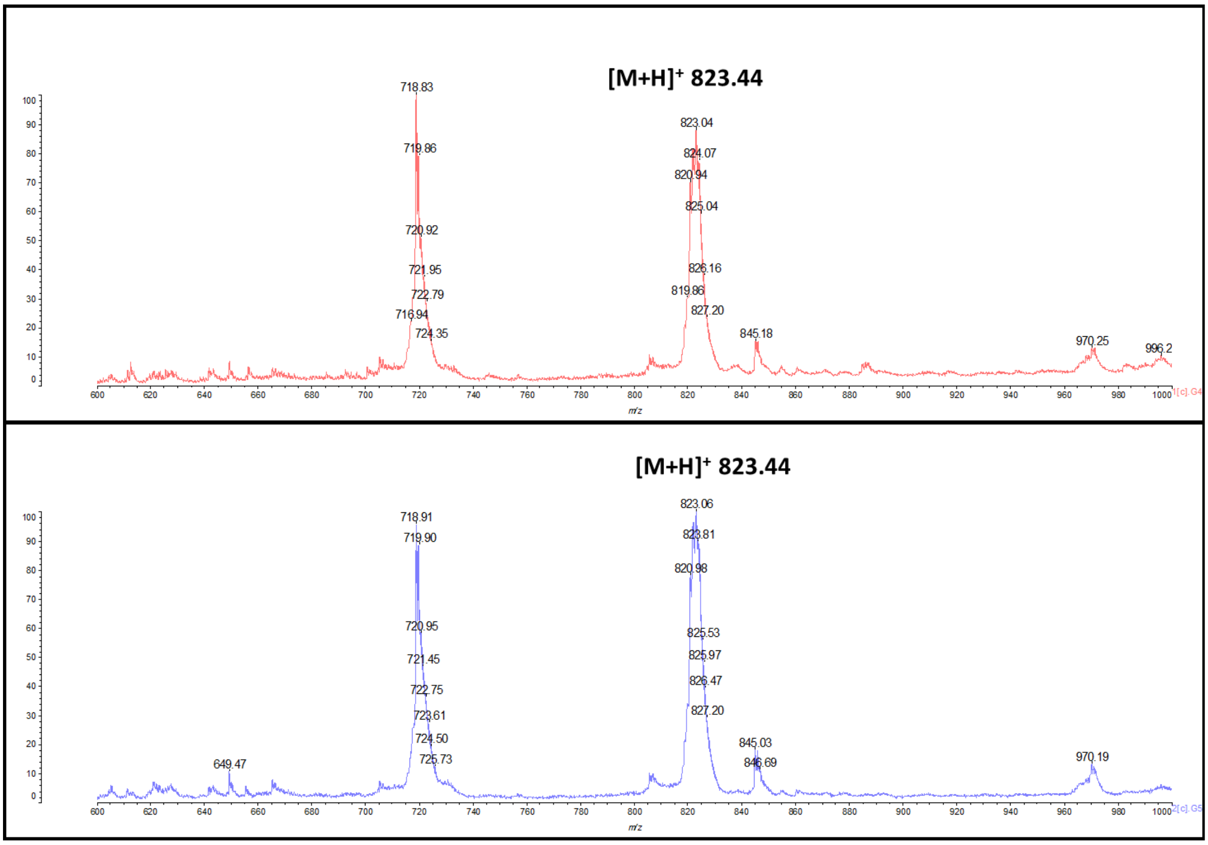

Supplementary Figure 4. MALDI-TOF spectra of 1-R (red line) and 1-S (blue line).

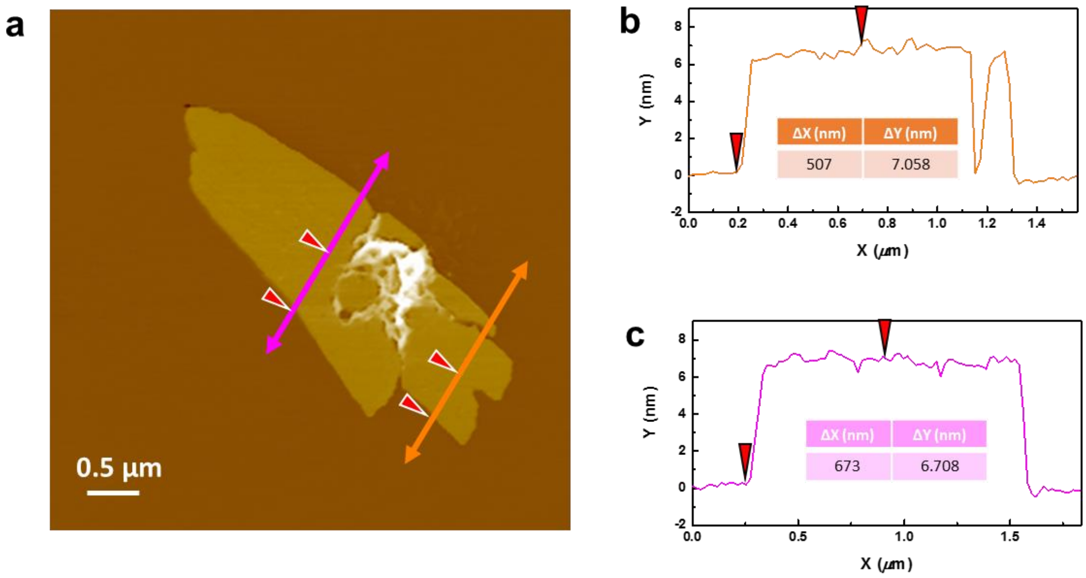

Supplementary Figure 5. a, AFM image of a nanoribbon intermediate obtained from self-assembly of 1-R. **b**,**c**, Cross sectional height profiles of the nanoribbon.

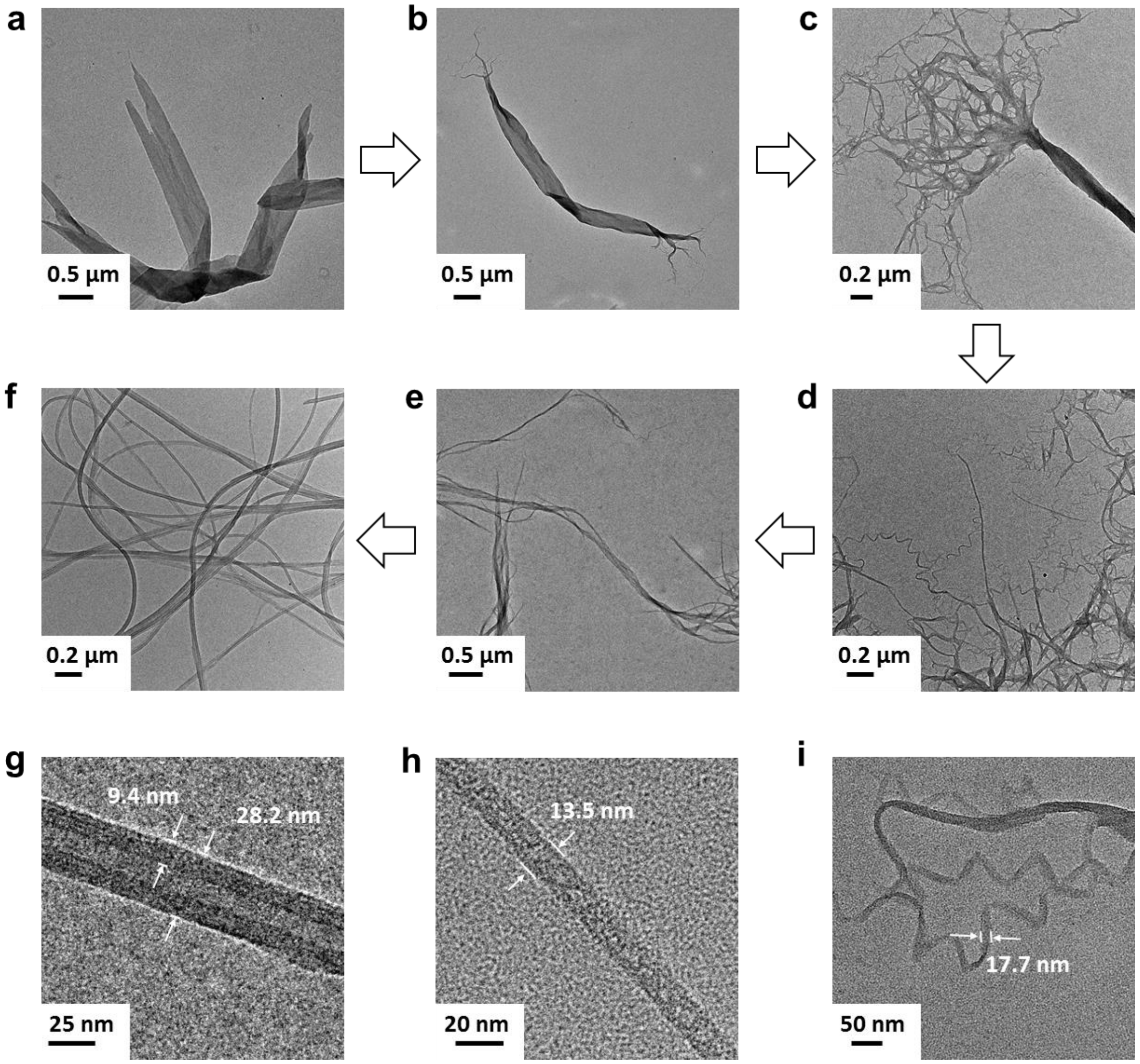

Supplementary Figure 6. Time evolution of 1-S from nanoribbon to nanotube. a-f, TEM images showing the transformation from nanoribbons to nanotubes during self-assembly of 1-S. The initially formed nanoribbons (**a**) (5 min) begin to develop split-ends (**b**) (10 min) and dissociate further (**c**) (30 min) to form nanocoils (**d**) (1 h), which are transformed to twisted nanofibers (**e**) (3 h) and eventually to nanotubes (**f**) (5 h). **g-i**, TEM images of individual nanotube (**g**), nanohelix (**h**) and nanocoil (**i**). Self-assembly condition: 27 µM of 1-S in 10% $CHCl_3$/EtOH, 65 ^{o}C (clear solution) to 15 ^{o}C in a temperature controlled cell, cooling rate: 10 ^{o}C/min.

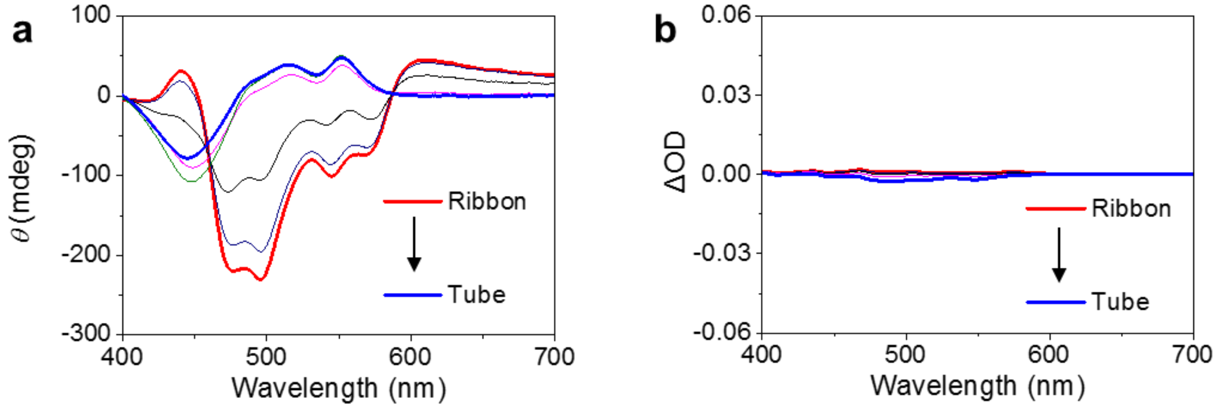

Supplementary Figure 7. Time-dependent changes in CD (**a**) and linear dichroism (LD) (**b**) spectra of 10% $CHCl_3$/EtOH solutions (27 μM) of 1-R obtained by heating at 65 ºC followed by cooling at 15 ºC (0 to 5 h).

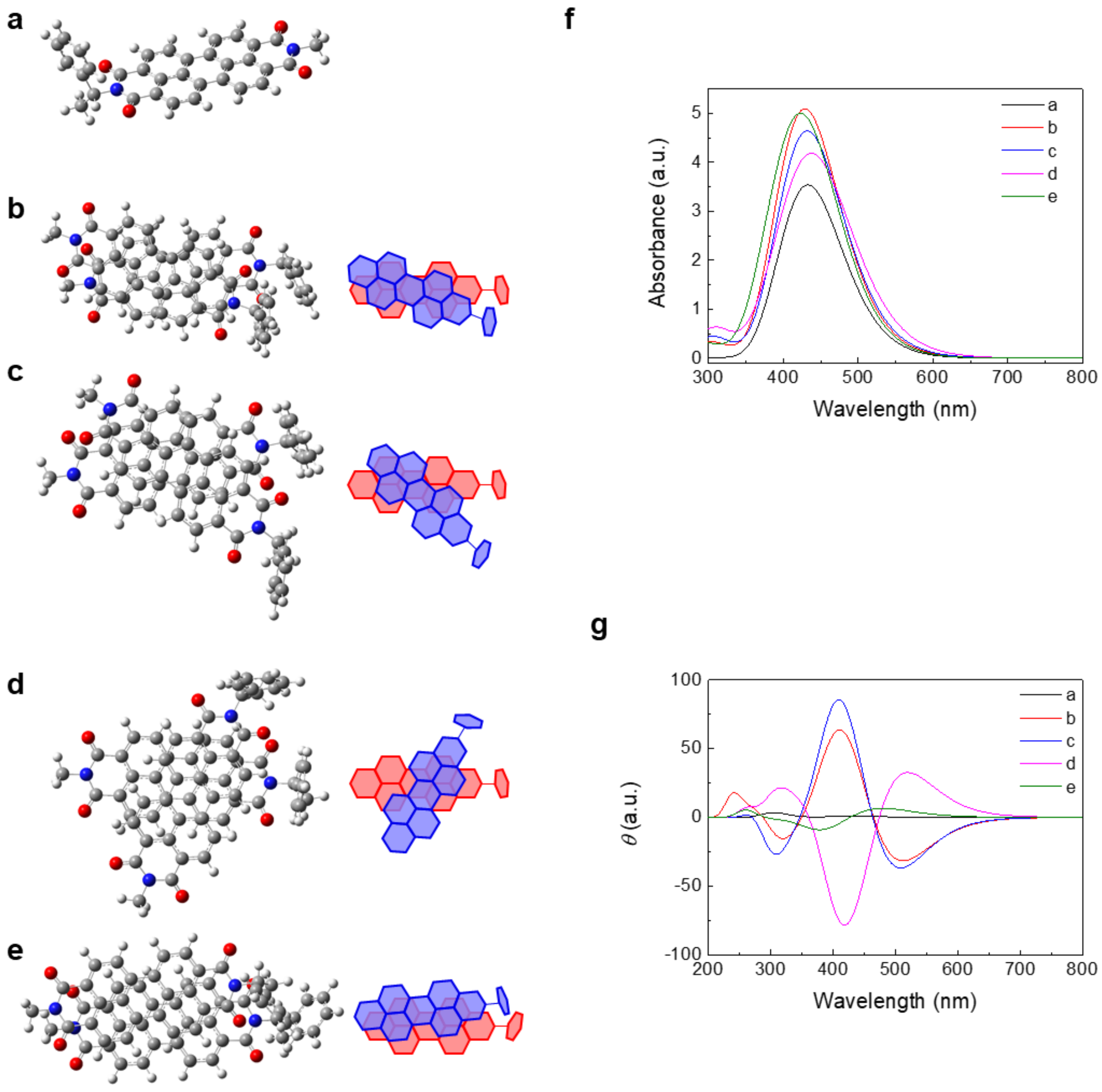

Supplementary Figure 8. **Quantum chemical calculation of 1-R**. **a**, Model molecule of **1-R**. **b**,**c**, Left-handed dimers of model molecule of **1-R**. **d**,**e**, Right-handed dimers of model molecule of **1-R**. **f**, Absorption spectra of model molecule of 1-R. **g**, CD spectra of model molecule of **1-R**. Left-handed (**b**, **c**) and right-handed (**d**, **e**) dimers exhibit negative and positive Cotton effects in CD spectra, respectively.

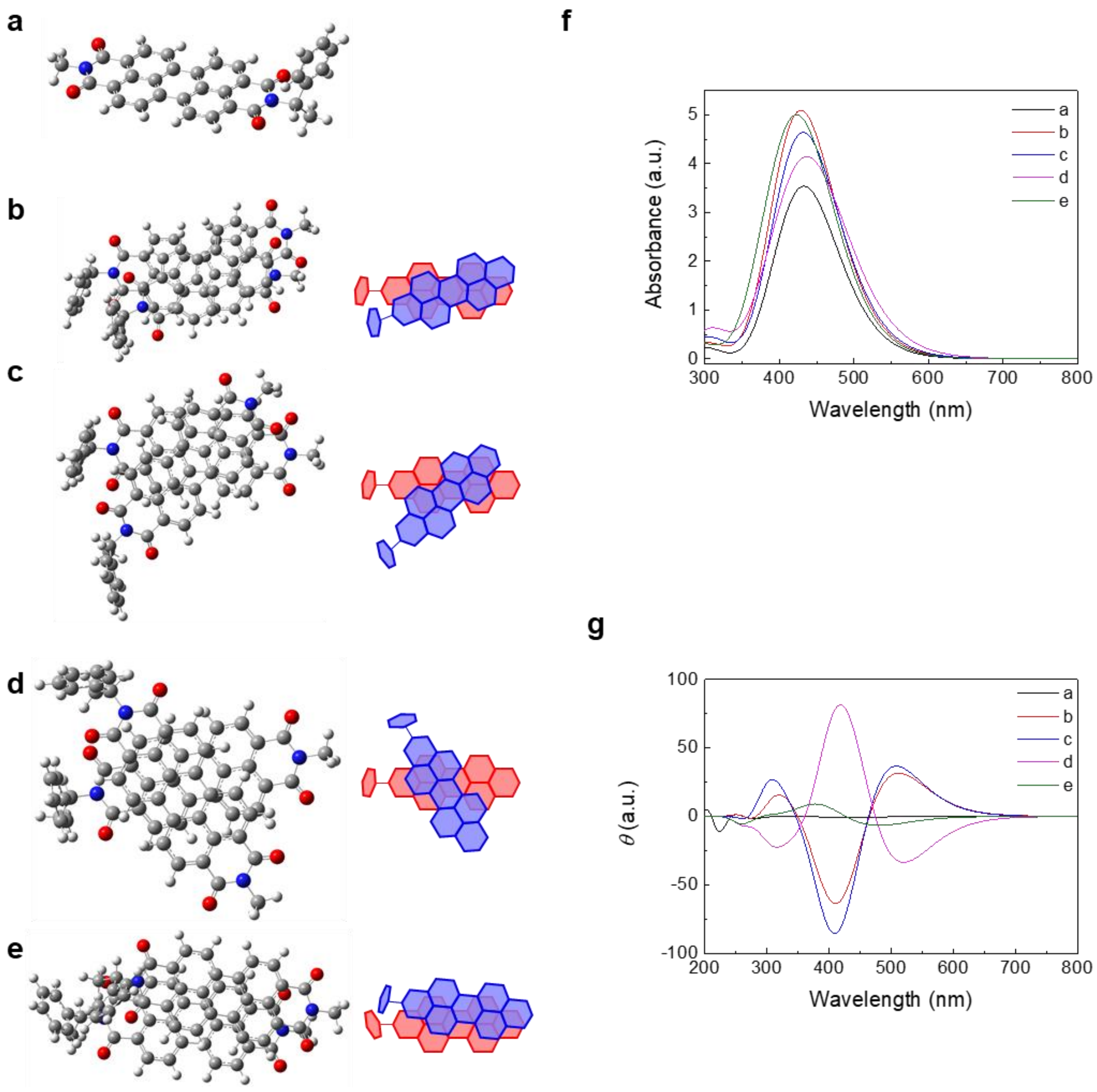

Supplementary Figure 9. **Quantum chemical calculation of 1-S**. **a**, Model molecule of 1-S. **b**,**c**, Left-handed dimers of model molecule of 1-S. **d**,**e**, Right-handed dimers of model molecule of 1-S. **f**, Absorption spectra of model molecule of 1-S. **g**, CD spectra of model molecule of 1-S. Left-handed (**b**, **c**) and right-handed (**d**, **e**) dimers exhibit positive and negative Cotton effects in CD spectra, respectively.

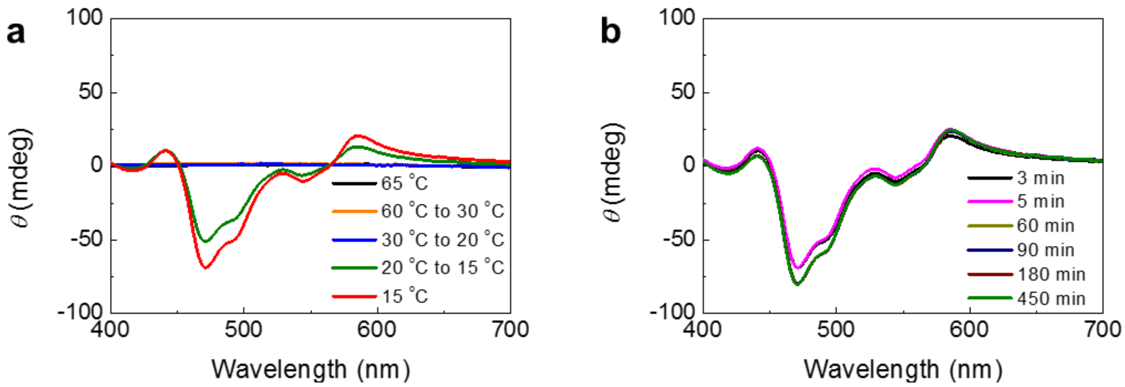

Supplementary Figure 10. a, **b**, Temperature (**a**) and time (**b**)-dependent CD spectral changes of a 1:1 mixture of 1-R and 1-S in 10% $CHCl_3$/EtOH solution (27 μM).

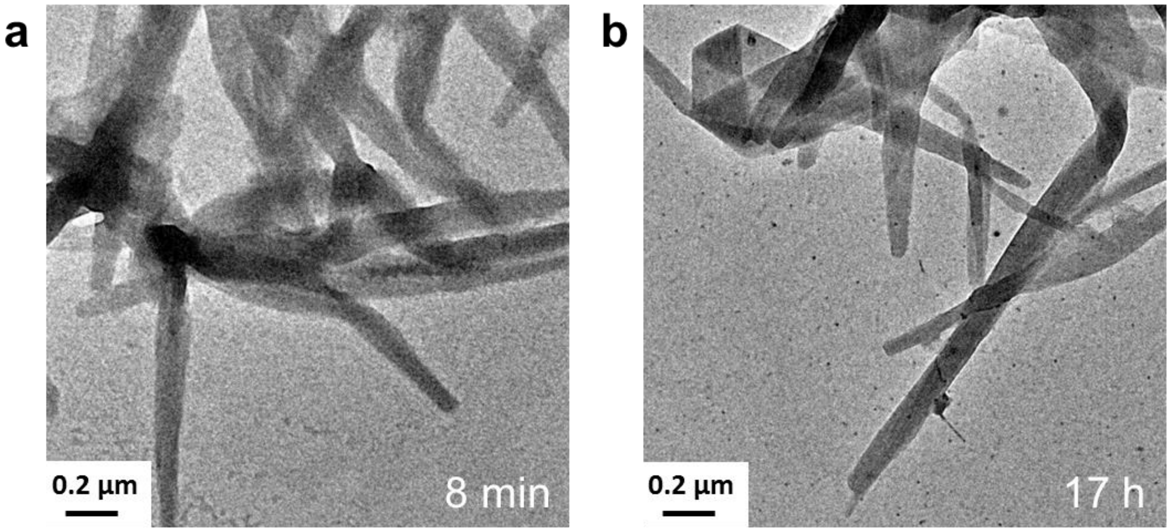

Supplementary Figure 11. TEM images obtained after 8 min (**a**) and 17 h (**b**) with a 1:1 mixture of 1-R and 1-S in 10% $CHCl_3$/EtOH solution (27 μM) at 15 ºC. Self-assembly condition: heating the solution to 65 ºC (clear solution) and cooling to 15 ºC in a temperature controlled cell, cooling rate: 10 ºC/min.

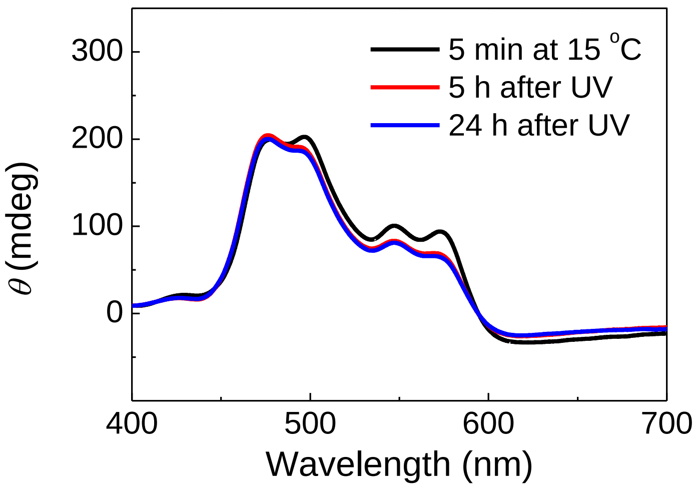

Supplementary Figure 12. CD spectra of 10% $CHCl_3$/EtOH solutions (27 μM) of and 1-S obtained by heating at 65 °C followed by cooling at 15 °C for 5 min (black), and then UV light irradiation (254 nm, 25 mW cm^{-2}, 10 sec) and standing at 15 °C for 5 h (red line) and 24 h (blue line), respectively.

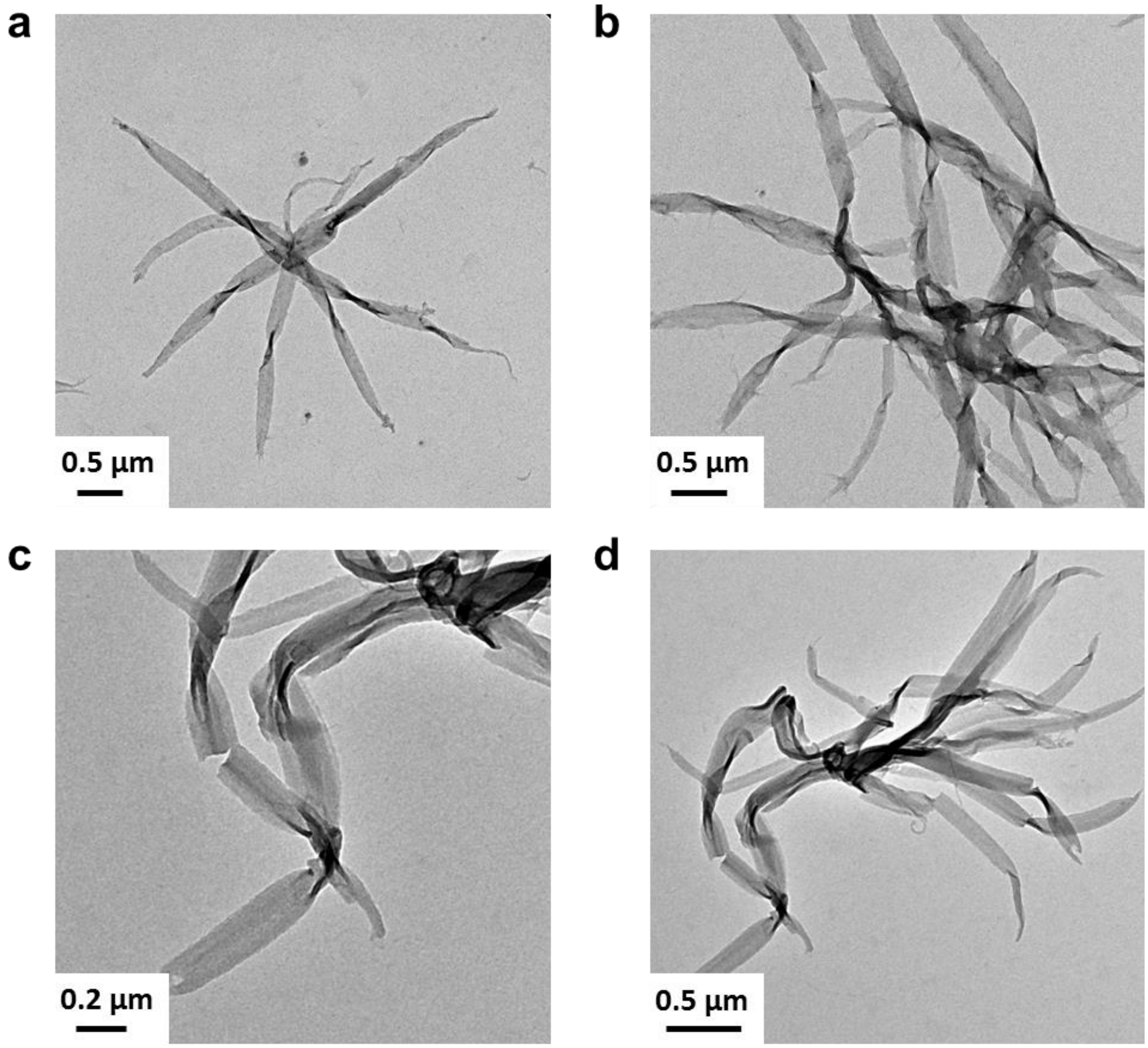

Supplementary Figure 13. TEM images obtained after 24 h of the UV-irradiated (254 nm, 25 mW cm^{-2}, 10 s) of 1-R (**a-b**) and 1-S (**c-d**) solutions that containing nanoribbons. Self-assembly condition: 27 μM of 1-S or 1-R in 10% $CHCl_3$/EtOH, 65 ºC (clear solution) to 15 ºC in a temperature controlled cell, cooling rate: 10 ºC/min.

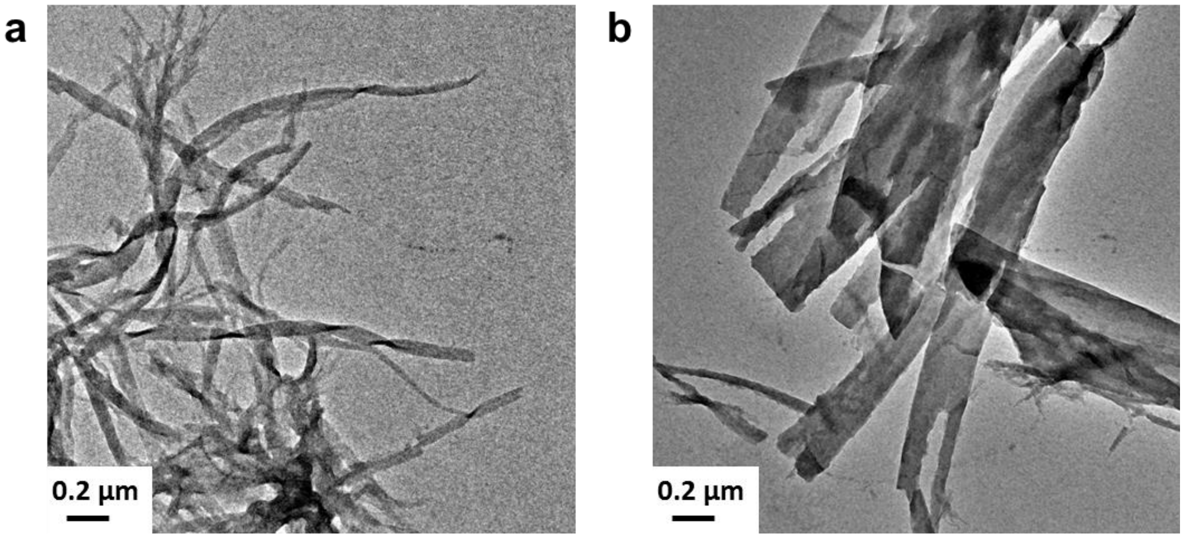

Supplementary Figure 14. TEM images obtained after 3 (**a**) and 5 (**b**) months at room temperature of the UV-irradiated (254 nm, 25 mW cm^{-2}, 10 s) 1-R (**a**) and 1-S (**b**) solutions that containing nanoribbons. Self-assembly condition: 27 μM of 1-S or 1-R in 10% $CHCl_3$/EtOH, 65 ºC (clear solution) to 15 ºC in a temperature controlled cell, cooling rate: 10 ºC/min.

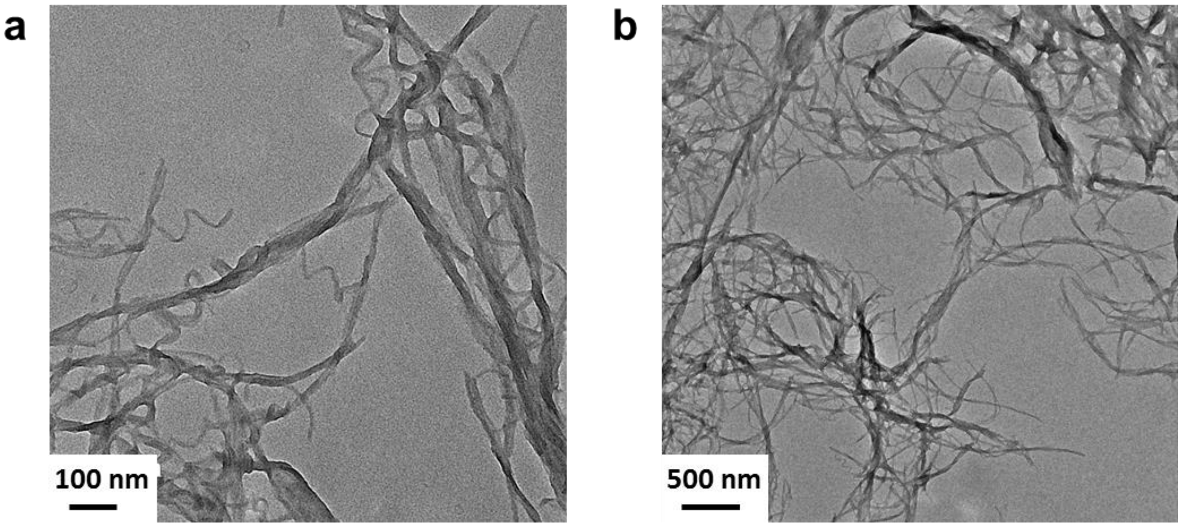

Supplementary Figure 15. a,**b**, TEM images UV-irradiated 1-R solution obtained by heating at 65 ^{o}C followed by cooling at 15 ^{o}C for 3 h and then UV light irradiation (254 nm, 25 mW cm^{-2}, 10 sec) and standing at 15 ^{o}C for 24 h.

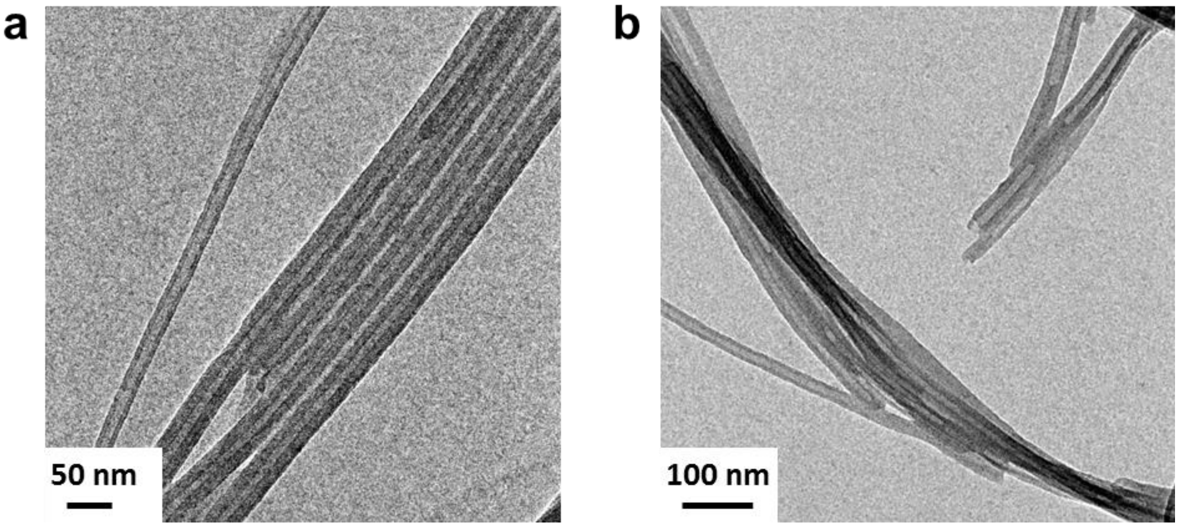

Supplementary Figure 16. a,**b**, TEM images UV-irradiated 1-R solution obtained by heating at 65 ^{o}C followed by cooling at 15 ^{o}C for 5 h and then UV light irradiation (254 nm, 25 mW cm^{-2}, 10 sec) and standing at 15 ^{o}C for 24 h.

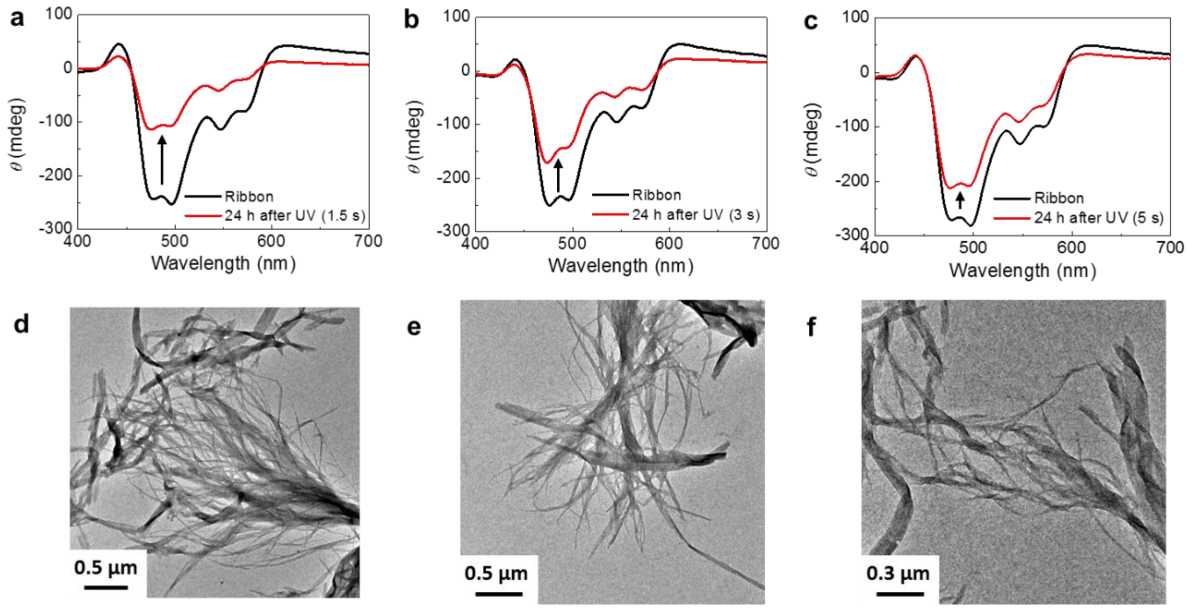

Supplementary Figure 17. a-c, CD spectra of 10% $CHCl_3$/EtOH solutions (27 µM) of 1-R obtained by heating at 65 °C followed by cooling at 15 °C for 5 min (black line). Red lines represent the CD spectra obtained after UV light irradiation (254 nm, 25 mW cm^{-2}) for 1.5 sec (**a**), 3 sec (**b**), 5 sec (**c**) followed by standing at 15 °C for 24 h. **d-f,** TEM images of 1-R aggregates obtained after UV-irradiation (254 nm, 25 mW cm^{-2}) for 1.5 sec (**d**), 3 sec (**e**) and 5 sec (**f**) followed by standing at 15 °C for 24 h.

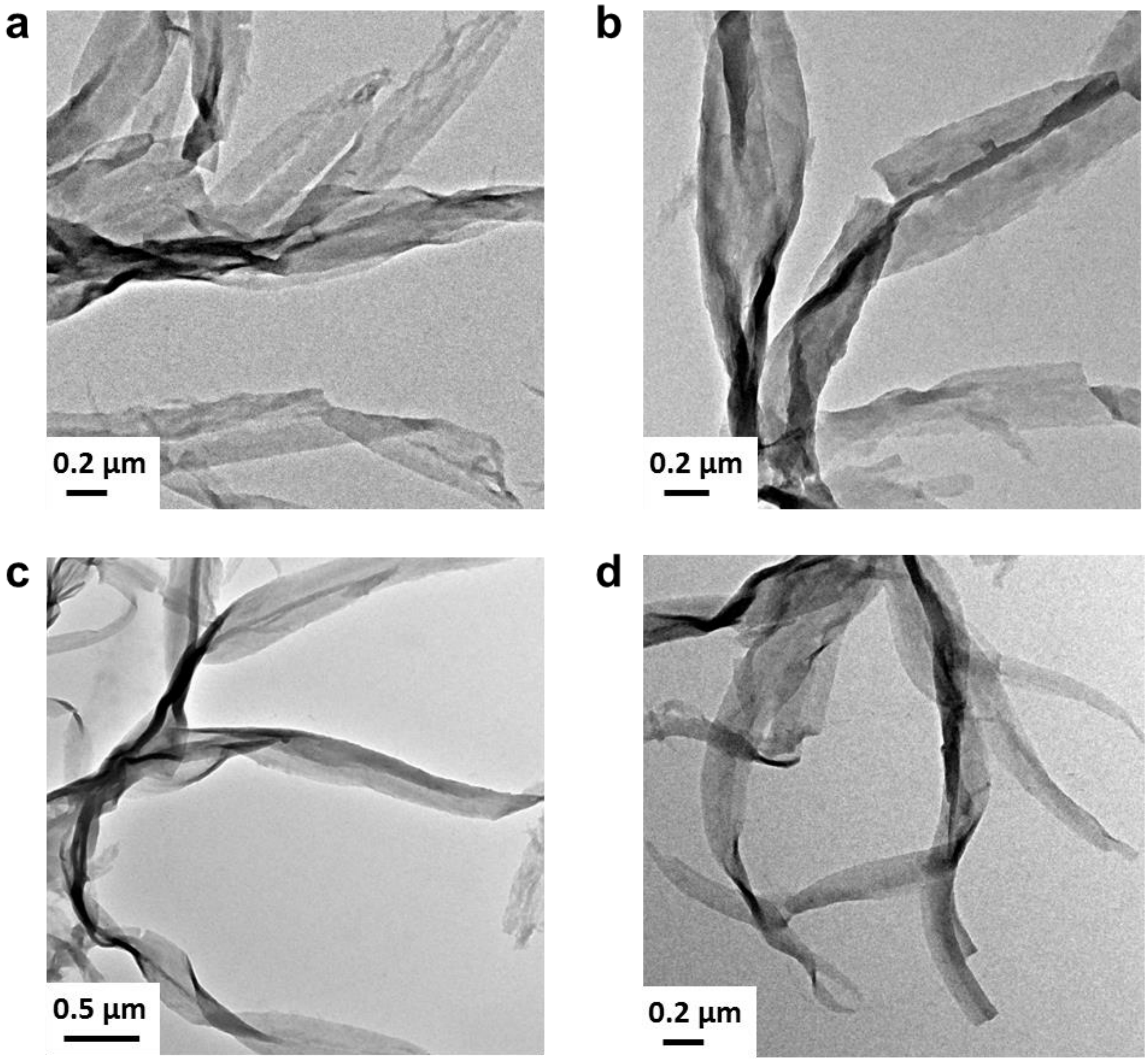

Supplementary Figure 18. TEM images of 1-R (**a**,**b**) and 1-S (**c**,**d**) nanoribbon obtained after UV-irradiation (254 nm, 25 mW cm^{-2}, 10 s) followed by heat treatment at 65 ºC for 10 min. Self-assembly condition: 27 µM of 1-S or 1-R in 10% $CHCl_3$/EtOH, 65 ºC (clear solution) to 15 ºC in a temperature controlled cell..

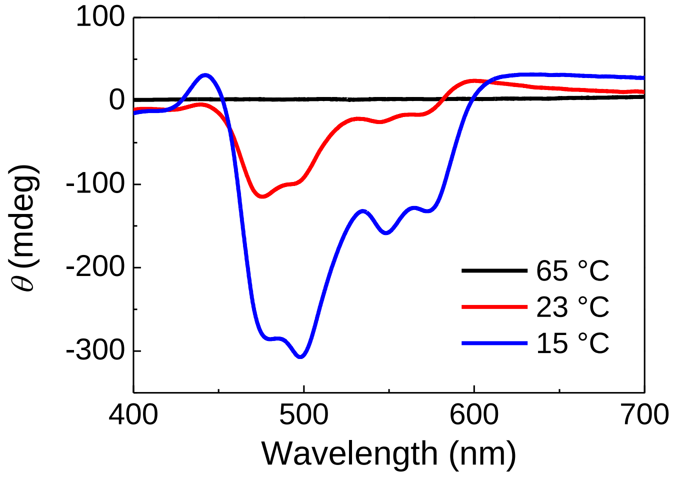

Supplementary Figure 19. CD spectrum (black line) obtained after heating a solution of 1-R at 65 ^{o}C followed by cooling for 5 h at 15 ^{o}C, and then heating at 65 ^{o}C. The solution is then cooled to 23 ^{o}C (red line) and 15 ^{o}C (blue line). The disappearance of the CD signal at 65 ^{o}C (black line) is caused by regeneration of unassembled 1-R.

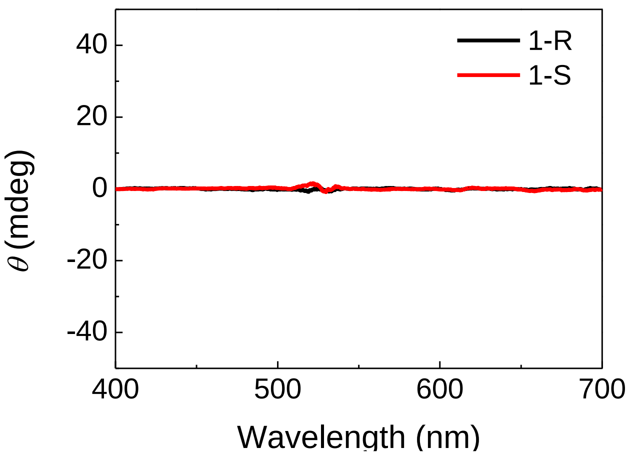

Supplementary Figure 20. CD spectra 1-R (black line) and 1-S (red line) in $CHCl_3$ solution (27 μM) at 65 °C.

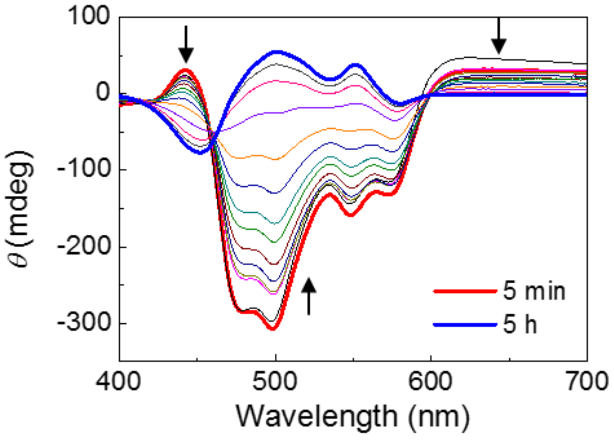

Supplementary Figure 21. Time-dependent CD spectral changes of 1-R in 10% $CHCl_3$/EtOH solution (27 μM) containing nanoribbons which were obtained by a heating (65 ºC)-cooling (15 ºC) process.

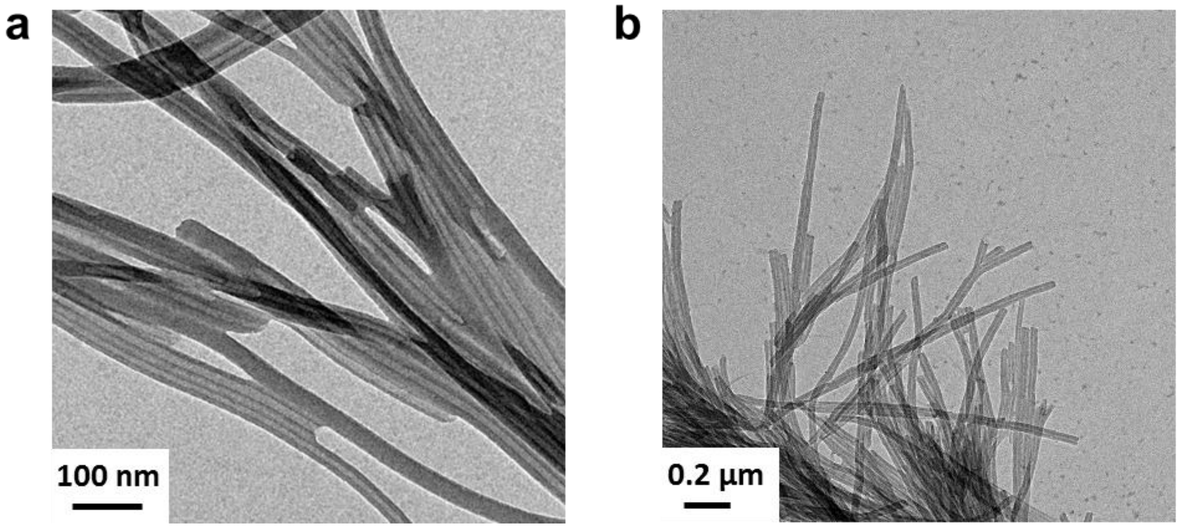

Supplementary Figure 22. TEM images of 1-R (**a**) and 1-S (**b**) nanotube solution obtained after UV-irradiation (254 nm, 25 mW cm^{-2}, 10 s) followed by heat treatment at 65 ºC for 10 min. Self-assembly condition: 27 μM of 1-R or 1-S in 10% $CHCl_3$/EtOH, 65 ºC (clear solution) to 15 ºC in a temperature controlled cell, stand the solution for 5 h at 15 ºC.

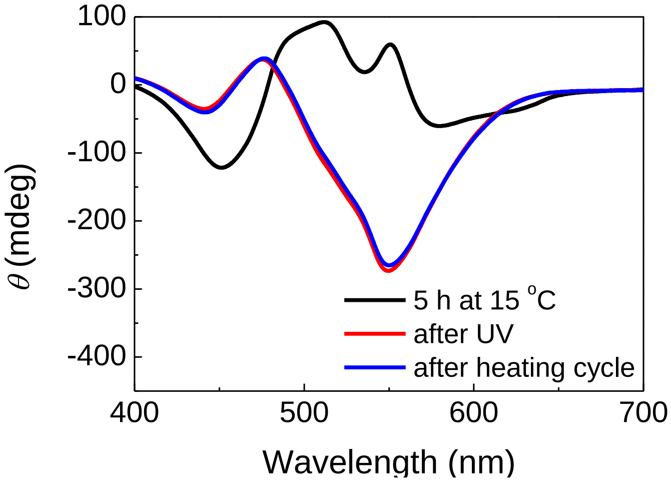

Supplementary Figure 23. CD spectrum of a nanotubular solution of 1-R (black line). The red and blue lines represent CD spectra of the UV irradiated (254 nm, 25 mW cm^{-2}, 10 sec) 1-R tubular solution before (red line) and after (blue line) heating cycle (15 °C-65 °C-15 °C).

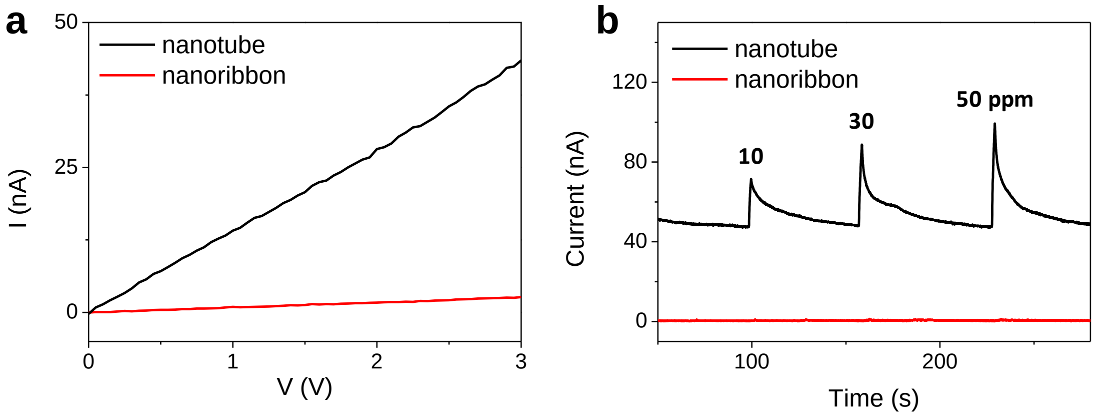

Supplementary Figure 24. **a**, **b**, Photocurrent as a function of applied voltage (**a**) and photocurrent responses upon exposure to various concentrations (10-50 ppm) of NH_3 gas (**b**) of UV-polymerized nanoribbon and nanotube forms of 1-R. Samples (ca. 100 μL) of 1-R solutions, obtained by UV-irradiated (254 nm, 25 mW cm^{-2}, 10 sec) at 5 min (nanoribbons) and 5 h (nanotubes) after initiation of self-assembly at 15 °C, were dropcasted between two electrodes and the solvent was removed by evaporation**.**

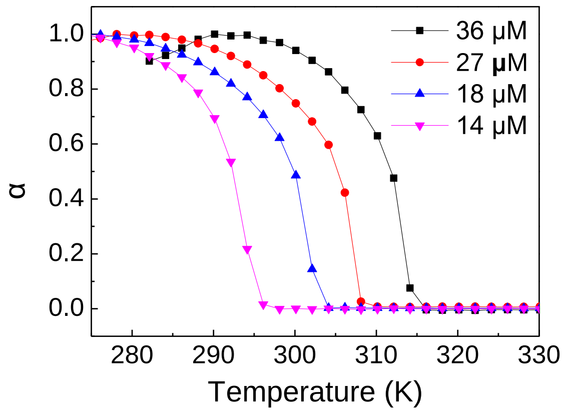

Supplementary Figure 25. Degree of aggregation (α) of different concentrations of nanoribbon of 1-R as a function of temperature at 500 nm, respectively in 10% $CHCl_3$/EtOH solution. Degree of aggregation was calculated from measured molar circular dichroism at 500 nm.

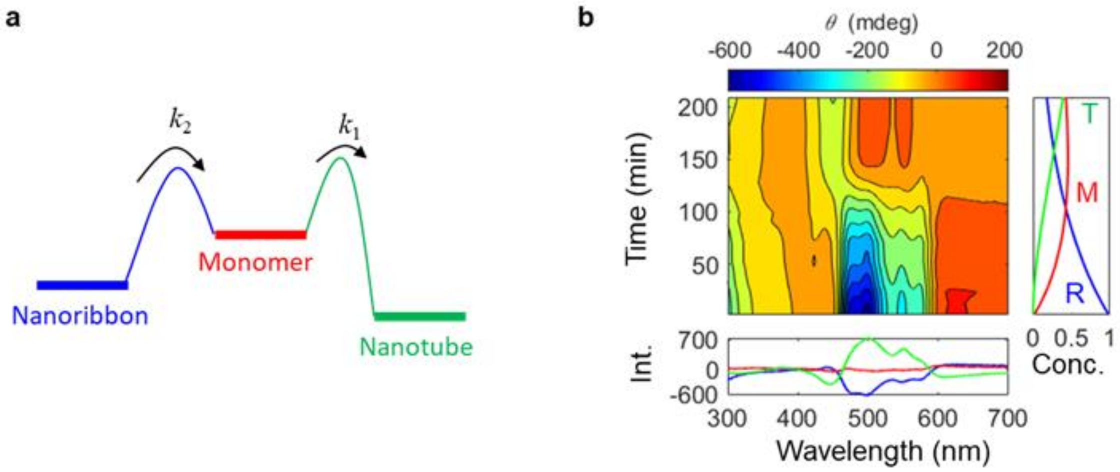

Supplementary Figure 26. **a**, The kinetic model of conversion processes from nanoribbon to nanotube. **b**, Global fitting analysis of time-dependent CD spectra of 1-R. The spectral components and time-dependent population decays of nanoribbon (R, blue), monomer (M, red), and nanotube (T, green) which are extracted from the global fitting analysis based on the kinetic model in **a**, are presented in the bottom and right panels, respectively. The rate constants are found to be k_1 = 121 min and k_2 = 191 min.

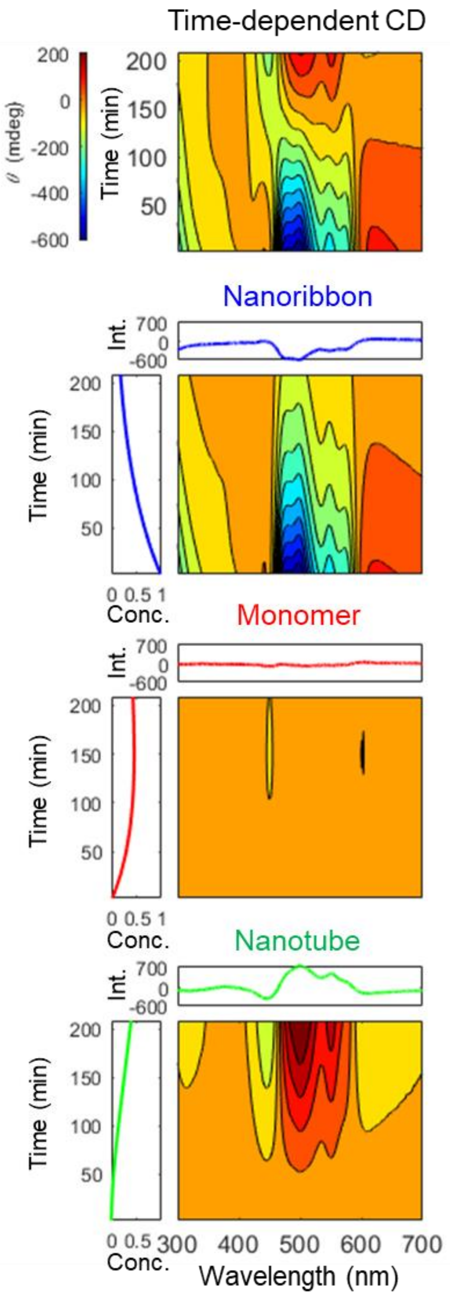

Supplementary Figure 27. Fitted time-dependent CD spectra and time-dependent concentrations of nanoribbon, monomer, and nanotube.

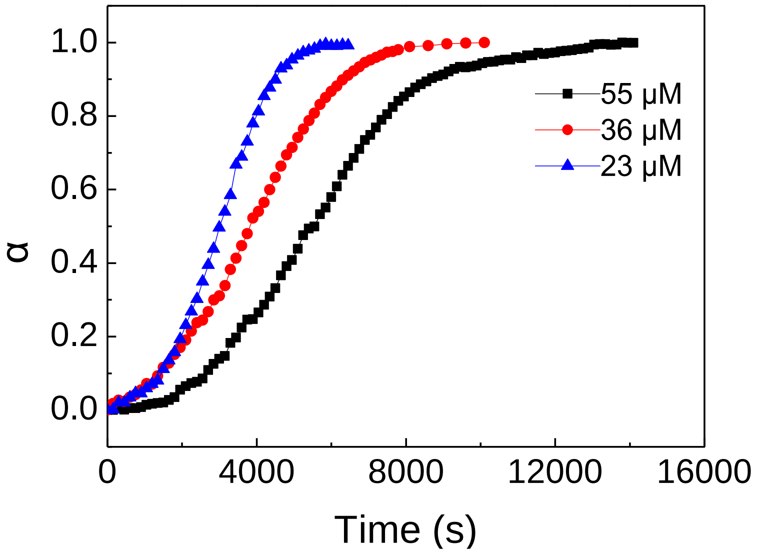

Supplementary Figure 28. Concentration-dependent transformation of 1-R nanoribbons into nanotubes. Plots of degree of aggregation (α), calculated form CD monitoring at 547 nm during transformation from nanoribbons to nanotubes as a function of time.

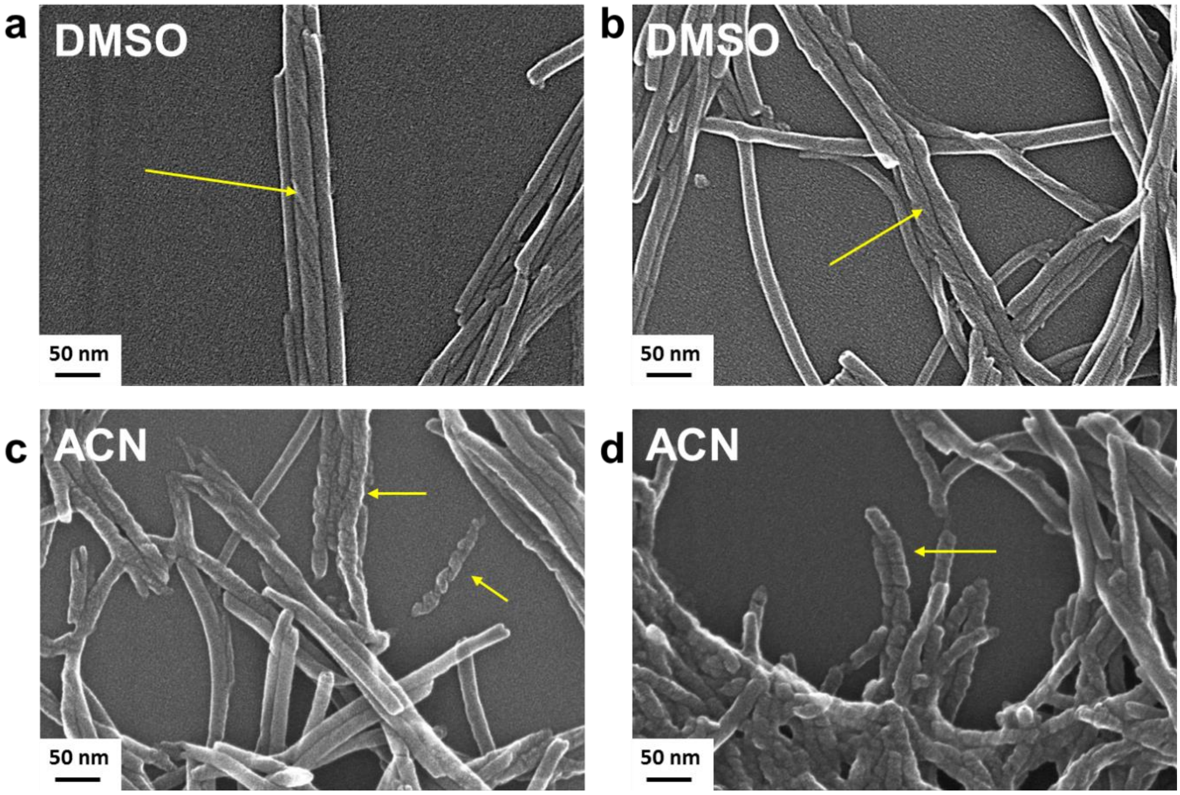

Supplementary Figure 29. TEM images of 1-R nanotube solution obtained after UV-irradiation (254 nm, 25 mW cm^{-2}, 10 s) followed by exposure to DMSO (**a**,**b**) and acetonitrile (ACN) (**b**,**c**). Yellow arrows indicate helical structures.

11. References

1. Jeong, W., Khazi, M. I., Lee, D. G. & Kim, J.-M. Intrinsically porous dual-responsive polydiacetylenes based on tetrahedral diacetylenes. *Macromolecules* **51,** 10312-10322 (2018).
2. Pasaogullari, N., Icil, H. & Demuth, M. Symmetrical and unsymmetrical perylene diimides: Their synthesis, photophysical and electrochemical properties. *Dyes Pigm*. **69,** 118-127 (2006).
3. Sheida, A., Duygu U. & Huriye, I. Chiral substituent containing perylene monoanhydride monoimide and its highly soluble symmetrical diimide: synthesis, photophysics and electrochemistry from dilute solution to solid state. *Photochem. Photobiol. Sci.* **7,** 936-947.
3. Chai, J. D. & Head-Gordon, M. Long-range corrected hybrid density functionals with damped atom-atom dispersion corrections. *Phys. Chem. Chem. Phys.* **10,** 6615-6620 (2008).
4. McLean, A. D. & Chandler, G. S. Contracted Gaussian basis sets for molecular calculations. I. Second row atoms, Z=11–18. *J. Chem. Phys.* **72,** 5639-5648 (1980).
5. M. J. Frisch. et al. Gaussian 16, Revision B.01 (Gaussian, Inc., Wallingford CT, 2016).
6. Garrido, M., Rius, F. X. & Larrechi, M. S. Multivariate curve resolution-alternating least squares (MCR-ALS) applied to spectroscopic data from monitoring chemical reactions processes. *Anal. Bioanal. Chem.* **390,** 2059-2066 (2008).
7. van Wilderen, L. J., Lincoln, C. N. & van Thor, J. J. Modelling multi-pulse population dynamics from ultrafast spectroscopy. *PloS one* **6,** e17373 (2011).
8. Ruckebusch, C., Sliwa, M., Pernot, P., de Juan, A. & Tauler, R. Comprehensive data analysis of femtosecond transient absorption spectra: A review. *J. Photoch. Photobio. C* **13,** 1-27 (2012).
9. Joung, J. F., Kim, S. & Park, S. Effect of NaCl salts on the activation energy of excited-state proton transfer reaction of coumarin 183. *J. Phys. Chem. B* **119,** 15509-15515 (2015).
10. Baek, J. et al. Origin of the reversible thermochromic properties of polydiacetylenes revealed by ultrafast spectroscopy. *J. Phys. Chem. Lett.* **7,** 259-265 (2016).
11. Korevaar, P. A. et al. Pathway complexity in supramolecular polymerization. *Nature* **481,** 492-496 (2012).
12. Korevaar, P. A., de Greef, T. F. A. & Meijer, E. W. Pathway Complexity in π-Conjugated Materials. *Chem. Mater.* **26**, 576-586 (2013)
